# GBC: a parallel toolkit based on highly addressable byte-encoding blocks for extremely large-scale genotypes of species

**DOI:** 10.1186/s13059-023-02906-z

**Published:** 2023-04-17

**Authors:** Liubin Zhang, Yangyang Yuan, Wenjie Peng, Bin Tang, Mulin Jun Li, Hongsheng Gui, Qiang Wang, Miaoxin Li

**Affiliations:** 1 Program in Bioinformatics, Zhongshan School of Medicine and The Fifth Affiliated Hospital, Sun Yat-Sen University, Guangzhou, 510080 China; 2 Center for Precision Medicine, Sun Yat-Sen University, Guangzhou, China; 3 Center for Disease Genome Research, Sun Yat-Sen University, Guangzhou, China; 4 School of Medical Technology and Information Engineering, Zhejiang Chinese Medical University, Hangzhou, China; 5 The Province and Ministry Co-Sponsored Collaborative Innovation Center for Medical Epigenetics, Tianjin Medical University, Tianjin, China; 6 Behavioral Health Services, Henry Ford Health, Detroit, MI USA; 7 Center for Health Policy & Health Services Research, Henry Ford Health, Detroit, MI USA; 8 Mental Health Center, West China Hospital, Sichuan University, Chengdu, China; 9 Key Laboratory of Tropical Disease Control (SYSU), Ministry of Education, Guangzhou, 510080 China; 10 Guangdong Provincial Key Laboratory of Biomedical Imaging and Guangdong Provincial Engineering Research Center of Molecular Imaging, The Fifth Affiliated Hospital, Sun Yat-sen University, Zhuhai, China

**Keywords:** Large-scale genotypes, Genotype compression, Highly addressable genotype blocks, Byte-encoding genotypes, Genotype management, Parallelization algorithm, Cloud computation

## Abstract

Whole -genome sequencing projects of millions of subjects contain enormous genotypes, entailing a huge memory burden and time for computation. Here, we present GBC, a toolkit for rapidly compressing large-scale genotypes into highly addressable byte-encoding blocks under an optimized parallel framework. We demonstrate that GBC is up to 1000 times faster than state-of-the-art methods to access and manage compressed large-scale genotypes while maintaining a competitive compression ratio. We also showed that conventional analysis would be substantially sped up if built on GBC to access genotypes of a large population. GBC’s data structure and algorithms are valuable for accelerating large-scale genomic research.

**Supplementary Information:**

The online version contains supplementary material available at 10.1186/s13059-023-02906-z.

## Background

Interrogating the full genetic spectrum underlying phenotypes in species requires large samples. With the dramatic decrease in sequencing costs and advancements in precision medicine, genome-wide genotypes of millions of subjects will soon become routinely available. However, the storage and computational demands of managing large-scale genotypes have become increasingly challenging. The Variant Call Format (VCF) [1] is a widely used framework to store the aggregate information of genotypes for genome sequencing projects, and it has become the standard format for genetic and genomic studies. However, the easy-to-read text format of VCF files occupies much redundant storage space and is not specifically designed for large-scale genotype data. Access to large-scale genotypes in VCF for analyses will impose a huge computational burden, usually due to data overload. While a binary format by PLINK was proposed to store large-scale genotypes efficiently and speed up genome-wide association studies (GWASs) [2], it loses information for multi-allele genotypes and has no further compression and addressing strategies besides binary encoding. Thus, there is an urgent need for a more efficient data format and high-performance utilities to meet the demands of large genetics and genomics projects, such as the UK Biobank (UKBB) [3].

Multiple methods have been proposed to compress genotypes to facilitate the transfer and storage of large-scale genotype data, e.g., TGC [4], GTShark [5], Genozip [6], and VCFShark [7]. However, these methods did not support rapid and flexible queries for the compressed data, which is important for analyses. Thus, block-based methods for compressing genotypes into rapidly accessible formats were developed subsequently, including BCFTools [8], PBWT [9], BGT [10], GQT [11], and GTC [12]. Unfortunately, the block-based methods are still generally inefficient for extremely large-scale genotypes (say, whole-genome genotypes of millions of subjects) due to a lack of efficient structures and algorithms. Methods for such large-scale genotypes should have at least four major technical advantages. First, memory and time overhead usage should grow linearly or keep steady when compressing and accessing a larger scale of genotypes. Second, it should have a robust framework for massive parallelization in reading, analyzing, and outputting genotypes. Third, it should efficiently manage the compressed genotypes (e.g., merging, splitting, and sorting by coordinates of variants). Finally, it should be equipped with versatile data query and output functions, say, query by specified variants or subjects, the output of phased or unphased genotypes with various formats (e.g., text, binary, byte, or other specified formats). Note that some advantages may conflict with each other in performance. For example, memory usage and disk input-output (I/O) efficiency may become inefficient when there are many parallel tasks, entailing a robust design to achieve these advantages simultaneously.

In the present study, we first proposed a unified data structure, Genotype Block (GTB) format, to store large-scale genotypes into many highly addressable byte-encoding compression blocks. Then, multiple advanced algorithms and a parallel computing framework were developed for efficient compression, decompression, access, management, and analyses based on the GTBs. Finally, the format and algorithms were implemented into a user-friendly Genotype Block Compressor (GBC) toolkit. We demonstrated that GBC is much faster than alternative methods to access and manage genotypes in GTB. GBC follows the GA4GH [13] application program interface (API) specification (https://ga4gh-schemas.readthedocs.io/en/latest/schemas/variants.proto.html) for the design of Java structures for genetic variants. The GBC software package and API functions are publicly available at https://pmglab.top/gbc, which can be easily integrated into other tools and applied to various genomics projects.

## Results

### Overview of the GBC procedure

**Fig. 1:**
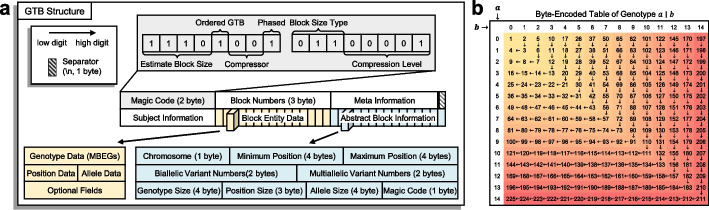
Structure of Genotype Block (GTB) and byte-encoded table of genotype (BEG) implemented in GBC. **a** Magic code: the first two bytes are used to store the compressed parameters. Block numbers: the total number of blocks contained in the compressed file, which also indicates that the “block abstract information” at the end of the file has (25*numbers) bytes of memory. Meta information: the meta information in the header of the VCF file. Subjects information: list of subjects. Block entity data: the compressed data is combined according to the order of the abstract block information. Abstract block information: abstract information of the GTB nodes for building the first-level fast index table. **b** The byte-encoding table of genotype (BEG)

**Fig. 2:**
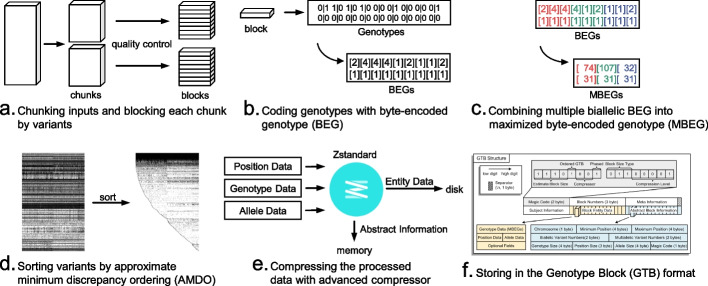
The workflow of building a GTB file in the GBC framework. **a** Slice (one or more) inputs into several chunks according to the specified number of parallel threads, and then each chunk is divided into several blocks for compression. **b** Code genotypes of each variant with byte-encoded genotype (BEG). **c** Combine multiple BEG of each biallelic variant into maximized byte-encoded genotype (MBEG). **d** Sort variants by approximate minimum discrepancy ordering (AMDO) to improve compression ratio. The sample graph is produced from the first 4000 biallelic variants of assoc.hg19.vcf.gz (download from https://doi.org/10.5281/zenodo.7737556). Genotype 0|0 is filled with white, 0|1 or 1|0 is filled with gray, and 1|1 is filled with black. **e** Compress the position, genotype, and allele data with an advanced compressor separately. Then, the compressed data (entity data) is concatenated into a long array and written into the disk, while the abstract information is recorded in the memory. **f** Store the compressed data in Genotype Block (GTB) format

We first designed a novel GTB format for compressing and storing large-scale genotype data of haploid or diploid species with various allele numbers, chromosome numbers, and phased or unphased genotypes (Fig. 1a). To begin the compression process, the input genotype file (in VCF format) is partitioned into several chunks and subsequently processed in parallel by multiple threads. In each parallel task, every chunk is further divided into multiple smaller indexable blocks. Here, a block is the smallest unit of compression, in which the number of variants is balanced with the sample size given the maximal array length ${2}^{31} -1(\approx 2\mathrm{GB})$ (Fig. 2a). Then, the genotypes of the variants are encoded into byte codes (Figs. 1b and 2b). For biallelic variants, their byte codes are further merged into one-byte codes by combining three phased or four unphased consecutive genotypes (Fig. 2c). Next, the approximate minimum discrepancy ordering (AMDO) algorithm is applied on the variant level (Fig. 2d) to sort the variants with similar genotype distributions for improving the compression ratio. The ZSTD algorithm is then adopted to compress the sorted data in each block (Fig. 2e). Finally, all the compressed blocks and metadata are written into a single GTB file (Fig. 2f). The procedure has a linear time complexity regarding the number of subjects and variants with small memory usage (less than 4 GB). It provides the fastest compression speed with a competitive compression ratio to date.

Besides, various advanced algorithms have been developed for rapid access and management of large-scale genotypes in GTB format. First, a tree structure (i.e., GTBTree) containing chromosome, block start, and end positions is designed for fast localization of and access to the indexable blocks and variants. Thus, data retrieval and editing in a large GTB file can be quickly accomplished only in involved blocks without decompressing the entire file. Second, an MBEG-based address conversion algorithm is developed for fast localization (especially for access by subjects) of a subject’s genotype at a variant, which enhances the speed of per-column access by more than 20 times (single thread, compared with BCFTools). Third, a unique sorting algorithm based on GTBTree makes it easy to sort variants of various sizes and degrees of disorder in less than 4 GB of memory by coordinates and without external disk space. Fourth, a file merging algorithm based on the minimum heap of sample sizes can quickly merge multiple large-scale genotypes in GTB format. Finally, we designed a cyclic locking model (CLM) for parallel decompression and downstream analysis, which can help users to design and perform parallelized computing efficiently. Thanks to the data structure and algorithms, GBC performs well for extracting, concatenating, merging, and splitting large-scale genotypes.

### Efficient compression of large-scale genotypes by GBC

**Fig. 3:**
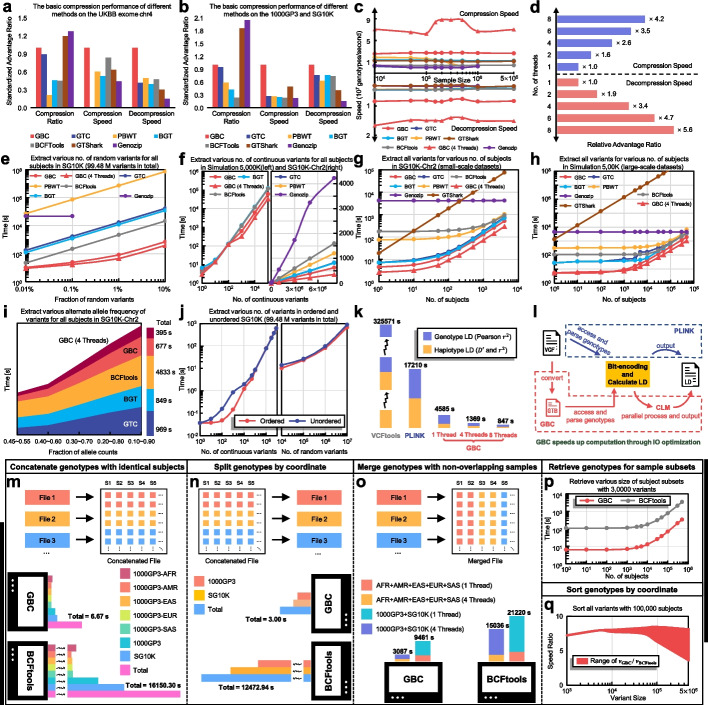
The performance comparison among GBC and alternative methods (details in Additional file 1: Tables S1-S5). **a** The basic performance of different methods on the UKBB exome chr4 dataset. The “standardized performance ratio” was obtained by scaling the GBC’s result to 1 and all other results against the GBC’s result. **b** The basic performance of different methods on the 1000GP3 and SG10K. **c** The compression speed (upper region) and decompression speed (lower region) of GBC and other alternative methods with the increase of sample size on simulated datasets. **d** The significant improvement of compression and decompression speed under multi-threads on the 1000GP3 dataset. **e** Retrieved the genotypes of random variants for all the subjects on the SG10K dataset. The option of accessing genotypes of variants in multiple regions at a time is only provided by BCFtools, GBC, and Genozip. Thus, the time cost was estimated by the access time of individual sites for methods including GTC, PBWT, and BGT. Genozip throws an exception when accessing 994,485 and 9,944,848 variants. **f** Retrieved a range of continuous variants for all the subjects on the Simulation 5000 K and SG10K-chr2 (the genotypes on chromosome 2 of the SG10K dataset) datasets. BGT, PBWT, GTC, and Genozip failed to compress the simulation 5000 K dataset. **g** Retrieved all the variants for a specified subset of subjects on the SG10K-chr2 dataset. **h** Retrieved all the variants for a specified subset of subjects on the Simulation 500 K dataset. **i** Filtered out the variants by alternative allele frequency on the SG10K-chr2 dataset. **j** Retrieved continuous variants and random variants in ordered and unordered SG10K dataset separately. **k** The comparison of LD coefficients computational speed between GBC and other popular tools on the 1000GP3 and SG10K datasets. **l** GBC speeds up follow-up computation (calculating the pair-wise linkage disequilibrium coefficients as an example) through I/O optimization. **m** Concatenate the chromosome-separated files within each dataset. **n** Split compressed archives by chromosome. **o** Merge multiple compressed archives with non-overlapping subjects. **p** Retrieve all the genotypes for a specified subset of subjects and rebuild the compressed archives. We tested the time cost of fetching different sizes of subject subsets on the simulation 500 K dataset. **q** Sort the variants by coordinate. We used several disordered simulated data with 100,000 subjects for evaluating the time cost and measured the range of speed ratio of GBC to BCFtools according to the disordered degree of datasets

A series of experiments were carried out to investigate the performance of the algorithms of GBC systematically. We first compared the compression ratio of the GBC with the other four widely used block-based methods for fast-accessing genotypes, including BCFTools, BGT, PBWT, and GTC. According to Danek et al. [12], GTC had the largest compression ratio among the four methods. However, Fig. 3a and b showed that GBC had the best compression ratio on the typical public datasets (UKBB, 1000GP3, and SG10K), over 10% higher than GTC. For example, GBC only needed 631.03 MB of disk space to store the UKBB dataset with 469,835 subjects and 70,581 variants, while GTC needed 701.45 MB. Besides, we also compared GBC to the other two genotype compression methods for data archiving (GTShark, Genozip). The two methods’ compression ratios were around 1.7 to 1.27 times higher than that of GBC for unphased genotypes (in UKBB exome chr4 dataset: GBC: 0.62 GB, GTShark: 0.51 GB, Genozip: 0.49 GB; in 1000GP3 and SG10K datasets: GBC: 3.68 GB, GTShark: 2.51 GB, Genozip: 2.54 GB, see details in Additional file 1: Table S1). However, accessing the compressed genotypes by the two methods is much slower than GBC (see details in the section about data-access performance). In sum, GBC provided a competitive compression ratio, although its primary goal is not to save the storage space of archived genotypes.

Then, we investigated the compression and decompression speed of the GBC using simulated and real genotypes. Figure 3c shows the compression and decompression speeds of GBC, as well as those of the alternative tools, on simulated datasets. We found that the GBC was much faster than GTC, which had the highest compression ratio among the four block-based genotype compression tools. The speed ratio of GBC to GTC grew exponentially as the sample size increased. For instance, GBC was over 1000 times faster than GTC in a simulated sample with 500,000 subjects. GBC took 0.80 min and 3.87 min to compress the genotype datasets with 30,000 variants of 100,000 and 500,000 subjects, respectively, while GTC spent 180 min and 4964.19 min doing this. In the real dataset testing, GBC was 454.73 times faster than GTC in compressing the UKBB [3] genotypes of 32,626 variants on chromosome 10 of 487,409 subjects. Besides, for the imputed genotypes of 4,562,904 variants on chromosome 10 of 487,409 UKBB subjects, GBC took 549.02 min (single-thread model) for the compression, while CTC failed after 3 weeks of running. Note that PBWT had memory overflow (> 100 GB memory), GTC had timeout (> 1 week) when compressing more than 500,000 subjects, and Genozip threw an exception when processing the datasets with 5,000,000 subjects. Compared to the two data-archiving methods (GTShark, Genozip), GBC was 2 to 5 times faster in compressing the testing datasets. This comparison showed that GBC had the superior speed to compress large-scale genotype datasets. Moreover, in terms of decompression speed, GBC was also approximately 1.8 times faster than others when decompressing the data to be BGZ format (Fig. 3c, lower region). Note that it is more reasonable to describe the speed difference in decompression with BGZ format, as it reduces the influence of the slow disk output speed. Finally, GBC can be several times faster under multi-threads for compression and decompression (Fig. 3d).

### Rapid accessing of the compressed genotypes by GBC

The time cost of accessing the compressed genotypes is very important for subsequent analyses. GTB format and GBC functions were primarily proposed to facilitate fast and flexible access to compressed genotypes. First, we compared the genotype access time of several alternative methods in extracting 0.01%, 0.1%, 1%, and 10% of the variants from the SG10K [14] dataset. We found that GBC was more than one order of magnitude times faster than others (Fig. 3e). Note that when retrieving multiple sporadic variants in a GTB file, GBC only needs to scan the file once, while PBWT, BGT, and GTC can only retrieve one variant in a file scan. Second, GBC was 2.07 times faster than the best of the other peer methods (i.e., BGT) when accessing a set of consecutive variants on the same chromosome (Fig. 3f). Moreover, GBC can also quickly retrieve the genotype data given a set of subjects. Its superior speed over other methods became increasingly striking as the sample size in compressed datasets increased. For example, when extracting genotypes in a single subject, GBC was 1.59 times faster than GTC on small-scale datasets, while it was 5.16 times faster on large-scale datasets (Fig. 3g, h). We indicated that all forms of data access would eventually approach the speed of decompressing all data as the number of genotypes to be extracted increases. However, GBC was still 1.8 times faster than the fastest method (GTC) on large sample sizes (Fig. 3c, lower region). Besides, GBC was also 1.25 times as fast as other methods when filtering out variants within a specified allele frequency range (Fig. 3i). We noted that Genozip and GTShark were much slower than GBC. For example, GTShark took 47,377.98 s to extract genotypes of a subject from a simulated genotype dataset of 100,000 variants of 5 million subjects, while GBC only needed 135.10 s. While GTShark only supports extracting genotypes of a single subject from the compressed dataset, Genozip can extract genotypes of multiple subjects or genotypes in multiple genomic regions from compressed datasets. However, in a simulated genotype dataset with 500,000 subjects and 30,000 variants, Genozip was 845.89 times slower than GBC in accessing the genotypes of a single subject (Fig. 3g, h, GBC’s 5.34 s vs Genozip’s 4518.75 s). Genozip was even 4576.7 times slower than GBC in accessing 9948 out of 99,448,478 variants in a dataset with 4810 real subjects in a test (Fig. 3e, GBC’s 10.27 s vs Genozip’s 46,998.60 s). Especially, GBC is by far the only method that enables fast retrieval in datasets with unsorted variants (while others only work for ordered variants). The time cost on the unordered datasets was similar to that of the ordered datasets (Fig. 3j). Last but not least, GBC is the tool that supports querying data in parallel. The query speed of GBC under multiple threads could be several times faster than that of a single thread (Fig. 3e-i), which suggests the great potential of GBC to accelerate parallel calculations.

### Significant improvement in the performance of downstream computation by GTB format

The genotype access and calculation can be carried out quickly and efficiently based on the GTB format, which speeds up follow-up analyses based on genotypes. Here, we showed the advantage of calculating the pair-wise linkage disequilibrium (LD) coefficients with GTB format. At the algorithmic level, GBC used bitwise operations to maximize computational speed, which is similar to PLINK [2] and KGGSeq [15]. However, GBC accessed genotypes for computation based on the GTB format rather than the vcf.gz format like other tools (such as PLINK, VCFtools [1], PopLDdecay [16]). Taking the 1000GP3 and SG10K datasets as an example, the results (Fig. 3k) showed that GBC was 3.75 times faster than PLINK(V1.9) in calculating LD from GTB data and compressed VCF data under a single thread, respectively. Especially, GBC was even 20.3 times faster than PLINK(V1.9) when both tools used eight threads. Here, we emphasized that the speed advantage of GBC is largely due to the faster genotype retrieving process and the cyclic-locked parallelization. Although the latest research indicated that GPU-based computing could significantly improve the speed of LD calculation [17], GBC mainly focused on optimizing the computational speed at the I/O level (Fig. 3l). The GTB format and GBC functions have largely relieved the limitation of I/O by optimizing the I/O frequency and load in genotype blocks. Therefore, we believe that many tools would perform better if they were built on GBC to access and parse large-scale genotype data.

### Convenient management of large-scale compressed files by GBC

Convenient management of large-scale compressed files is also critical for genetic and genomic studies. We compared GBC with the current popular tool BCFtools [8] for managing compressed genotype data (PBWT, BGT, and GTC do not support these functions). BCFtools handles compressed BCF in BGZ formats, while GBC manages compressed GTBs. When concatenating genotype files with the same number of subjects, we found that GBC was 2421.3 times (Fig. 3m) faster than BCFtools. When splitting genotypes by chromosome, it was 4157.6 times (Fig. 3n) as fast as BCFtools. Besides, GBC was 4.87 times faster than BCFtools on merging genotypes files with non-overlapping subjects under four threads (Fig. 3o). For retrieving the genotypes of 500,000 subjects, the speed of GBC was further increased by 5.8 times because of the advanced GTB structure (Fig. 3p) compared to the BGZ format (Fig. 3h). In terms of the sorting speed, GBC was always approximately 3 to 9 times faster than BCFtools for 100,000 subjects under a single thread (Fig. 3q). The less disordered the data, the better performance the GBC would have. Particularly, GBC can accomplish the sorting directly in 4 GB of memory, while the BCFtools may need large external disk space (Additional file 2: Fig. S1). For instance, BCFTools cannot sort larger datasets (e.g., 100,000 subjects and 1,000,000 variants) in 64 GB of memory. Similar to the data access performance, we believe that the speed difference between GBC and BCFtools will be more pronounced as sample sizes increase. These results illustrated that the GTB structure could also facilitate fast management of large-scale genotypes.

## Discussion

In recent years, numerous genome-wide genotypes of large-scale samples for dissecting genetic mechanisms of phenotypes have grown explosively, raising a heavy computational burden. Many methods were developed to address this issue by advanced algorithms compressing large-scale genotypes. However, efficient usage of large-scale genotype data is far more than compression. In the present study, we proposed a comprehensive solution for the management and computation of large-scale genotypes. First, we designed a unified data structure, GTB, to store any type of genotype data flexibly (phased or not, biallelic or multiallelic, human or non-human) into highly addressable byte-encoding blocks in a single file. We showed that the novel blocking structure facilitated rapid access to compressed genotypes compared to the existing genotype compression methods [9–12]. The single-file strategy also solved the management inefficiency of the multi-file strategy adopted by most compression methods [9–12]. Moreover, we developed multiple advanced algorithms based on the GTB format to improve the speed and save RAM for genotypes’ computation and management. All of them are implemented in our integrated software toolkit, GBC. To our knowledge, GBC may be the only tool that can compress and manage whole-genome genotypes of millions of human subjects in an ordinary computer with around 4 GB RAM. Furthermore, its I/O optimized framework for different types of parallel tasks (e.g., I/O intensive tasks and computing-intensive tasks) processing genotype blocks also enhance the efficiency of large-scale genotypes. Two commonly used and user-friendly interfaces are also provided, a command line tool (installation-free) and a Java-API library. Thus, GBC is suitable for a wide range of users, enabling professional researchers and amateurs to use it even on personal laptops easily.

The success of GBC showed that compression and rapid access of genotype data could be well balanced with subtle data structure and advanced algorithms. The data compression ratio and access speed interfere with each other. GBC does not aim to improve the compression ratio exclusively. Therefore, GBC may have a lower compression ratio than the methods for a frozen compression of genotypes which would be very inefficient for genotype access in subsequent analysis (e.g., GTShark [5], Genozip [6], VCFShark [7]). Besides the storage space, the speed of access and analysis is also critical for analyses with large-scale genotype data (e.g., UKBB [3] and TOPMed project [18]). GBC aims to improve both in a balanced way. While GBC achieves the fastest compression, decompression, query, and manipulation of compressed data among the tools that support fast retrieval, it also has a highly competitive compression ratio to the compared tools in real testing datasets. For example, among the four compared tools, GTC, the tool with the closest compression ratio to GBC, is much slower (more than 1283 times than GBC in compressing large-scale datasets with ~ 500,000 subjects). While some other tools are much faster than GTC, they are inferior to GBC in compression ratio. The GTB format and GBC functions have largely overcome the limitation of I/O by the I/O optimization. These are particularly beneficial for developing and applying genomic tools or platforms that analyze large-scale genotype data. For instance, sequential access to genotypes in GTB files is 5.78 times faster than that in vcf.gz format on 1000GP3, while random access to genotypes of a variant is estimated to be more than 71.56 times faster. Furthermore, the LD calculation speed has been improved by 20.3 times under eight threads due to the efficient I/O, compared to PLINK(V1.9).

It should be noted that the GTB format is not intended to replace VCF. VCF’s text format that denotes genotypes with various attributes is human-readable and convenient, making it widely used in the genetic and genomic community. Despite being inefficient for computing large-scale genotypes, VCF remains essential for sharing and exchanging called variants in the community. Nevertheless, GBC provides efficient functions to transform VCF to the GTB format, which has a much more efficient design to optimize access and computation of large-scale genotypes. GBC also includes a complete quality control procedure to ensure high-quality genotypes are compressed, with those failing to meet quality criteria being set as missing values. In the performance comparison of this paper, we removed all quality metrics to make a fair comparison with similar tools (which cannot store the quality metrics of genotypes, e.g., BGT [10] and GTC [12]). However, the flexible structure of GTB allows it to store extra data directly, including ID, INFO, and Genotype Metrics in VCF. This flexibility meets the general analysis requirements of a large-scale genotype dataset, which may include other data.

## Conclusions

Here, we developed a new toolkit, GBC, for efficient storage, access, management, and computation of extremely large-scale genotypes and verified its validity by various scales of genotypes. Most existing tools had no fast-accessible compression format and no efficient usage of RAM or I/O-optimized parallel computing for large-scale genotype data. In contrast, GBC provides a novel GTB data structure, speed-raising algorithms, and an I/O-optimized parallel framework with reusable RAM. The computing burden of GBC is so substantially reduced that whole-genome genotypes of millions of subjects can be rapidly compressed and accessed in small RAM under a fully parallelized model. We also showed that many commonly used software tools were very slow or required too much RAM to process the same amount of large-scale genotypes. Several extensions to GBC are under consideration for further development, including more efficient functions for large-scale genomic computations (e.g., the population PCA [19]) based on its parallelization and low memory consumption advantages. The ability of fast access to genotypes with low RAM consumption under the optimized parallel framework also makes GTB and GBC attractive for cloud computation. In sum, GTB and GBC will benefit genetics and precision medicine studies, whose genotype resources are growing dramatically.

## Methods

### Compress genotypes into a highly addressable Genotype Block (GTB) format

#### The structure of GTB

We designed a novel Genotype Block (GTB) format to store the compressed large-scale genotypes in a file. GTB consisted of 6 main decoupled parts (Fig. 1a, Additional file 3: Note 1). The first part is the magic code, which stores the software parameters for building the current GTB file using 2 bytes, including the maximum size of each block after decompression (for pre-allocated memory when decompressing blocks), whether the file is ordered or not, the specified compressor, the state of genotype (phased or unphased), the maximum number of sites per block, and the compression level. The second part is the block numbers, which stores the number of sub-blocks in the current GTB file as a 3-byte integer. The third part is the version of the reference genome for variants positions and so on (i.e., the meta-information in VCF). As an extension, other necessary comment fields in the VCF can be concatenated using an intermediate separator (e.g., “\t”) and stored in this part. The fourth section is the sample information, which contains the sample sequence length and the sample data. The fifth part is the genotype sub-blocks. Each sub-block contains three compulsory sub-parts, including genotype data, position data, and allele data. In addition, other fields from VCF can also be concatenated as optional sub-parts. Finally, the sixth part is the abstract block information, which contains the parameters necessary to access the genotype sub-blocks quickly (i.e., index information).

Here is a typical process for generating a GTB file. GBC first writes 5 bytes of placeholder information for magic code and block numbers at the beginning of compression. The meta information and subject information are added subsequently. Next, the data entity and abstract information for each block are written to the disk and memory synchronously to ensure that the order of records in memory is the same as those written to the file in the hard disk. After all the blocks are compressed, their abstract information in the memory is written to the end of the GTB file. Finally, the file pointer returns to the head of the file, and GTB modifies the placeholder information according to the compression results, including block size, compressor parameters, the state of genotype (phased or unphased), and whether the file is in order (whether all the block-coordinates in current GTB file are non-overlap). In brief, a GTB file is essentially made up of highly addressable and independent blocks of compressed genotypes.

#### Chunking inputs and splitting each chunk into blocks

The processed VCF file is sliced into $t$ chunks (if the multi-threading mode is enabled and $t$ threads are specified) with approximately even physical sizes. Under each thread, the read-in variants of each chunk are divided into multiple blocks of consecutive entries. Briefly, the block construction is terminated if (a) the number of variants in the block reaches the maximum size, (b) a different chromosome occurs, and (c) it reaches the end of the file. The maximum size of variants $N$ in each block is set automatically, which is mainly dependent on the sample size $M$ as $N\in \{{2}^{n}|{2}^{n}\times M\le {2}^{31}-2, 7\le n\le 14\enspace \mathrm{and}\enspace n\in {\mathbb{N}}^{*}\}$. GBC can store 128 variants of 16,777,215 subjects or 16,384 variants of 131,071 subjects in each block at most. This strategy allows the personal laptop to deal with large datasets conveniently because a block always occupies small memory during the analysis. The blocks from different chunks are parallelly processed independently under the multi-threaded mode, improving compression speed. Besides, we have adopted nine commonly used quality control strategies (described in KGGSeq [15]) in GBC to filter out genotypes and variants with poor quality. The quality control (QC) is executed by default after read-in, and only genotypes and variants that pass the QC are stored in the blocks. The blocks also allow the decompressing algorithm only to process a small fraction of all the data for queries (fast access to the compressed data).

#### Maximized byte-encoded of genotype (MBEG)

Conventional genotype array storage in text format (e.g., 0|1) takes up much redundant information space. Therefore, a more efficient and compatible strategy is needed to encode the genotypes with less space to enhance the information density (containing as much information as possible in the same byte length). Here, we propose a novel byte-based lossless encoding strategy to store genotypes, which helps reduce memory burden and accelerate the compression process. It has two steps, and step 2 is specifically designed for biallelic genotypes.

##### Step 1

A byte-based encoding strategy of genotype (byte-encoded genotype, BEG, Fig. 1b) is proposed to encode genotypes. Let a variant $v$ has ${n}_{v}$ different alleles ${n}_{v}\in [2, 15]$. The byte codes of a genotype of the variant can be calculated. The non-missing phased genotype “$a\mid b$” ($a, b \ge 0$) will be encoded as:

1 $$\begin{array}{c}a\mid b\to \left\{\begin{array}{ll}{\left(a+1\right)}^{2}-b &,a\ge b\\ {b}^{2}+a+1&,a<b\end{array}\right.\end{array}$$

The missing genotype “$.|.$”, is encoded to 0. For an unphased genotype “$a/b$”, it is also encoded formula (1) after being transformed to “$\mathrm{min}\{a, b\}\mid \mathrm{max}\{a, b\}$”. For the sake of unification, a genotype “$a$” of a variant in a male’s chromosome X and Y is converted into “$a\mid a$” and is encoded by the above formula. Note that the strategy ensures coherent genotype encoding values of variants with different allele numbers.

##### Step 2

However, the BEG array (BEGs) still has unused information space and may reduce the compression efficiency in a project with large samples. Because a biallelic variant has 4 (unphased) ~ 5 (phased) possible genotypes and the majority of variants in human genomes are biallelic, we further combine 3 (phased) ~ 4 (unphased) consecutive BEG codes into a single byte for biallelic variants as follow:

2 $$\begin{array}{c}\left[\!{\begin{array}{c}\text{BEG}\end{array}\!}_{0},{\begin{array}{c}\text{BEG}\end{array}\!}_{1},{\begin{array}{c}\text{BEG}\end{array}\!}_{2}\right]\to {5}^{2}\cdot {\begin{array}{c}\text{BEG}\end{array}\!}_{0}+5\cdot {\begin{array}{c}\text{BEG}\end{array}\!}_{1}+{\begin{array}{c}\text{BEG}\end{array}\!}_{2}\end{array}$$

3 $$\begin{array}{c}\left[\!{\begin{array}{c}\text{BEG}\end{array}\!}_{0},{\begin{array}{c}\text{BEG}\end{array}\!}_{1},{\begin{array}{c}\text{BEG}\end{array}\!}_{2},{\begin{array}{c}\text{BEG}\end{array}\!}_{3}\right]\to {4}^{3}\cdot {\begin{array}{c}\text{BEG}\end{array}\!}_{0}+{4}^{2}\cdot {\begin{array}{c}\text{BEG}\end{array}\!}_{1}+4\cdot {\begin{array}{c}\text{BEG}\end{array}\!}_{2}+{\begin{array}{c}\text{BEG}\end{array}\!}_{3} \end{array}$$

Conversions (2) and (3) are designed for phased and unphased BEGs, respectively. If the number of BEG for a variant is not enough to make a group of three or four, the inadequate part is called incomplete MBEG (e.g., one variant of 1000 subjects with phased genotypes will form 334 groups, and two nulls to make up the last group), and the null $${\begin{array}{c}\text{BEG}\end{array}}_{i}$$ will be set to the same as the previous one according to $${\begin{array}{c}\text{BEG}\end{array}}_{i}={\begin{array}{c}\text{BEG}\end{array}}_{i-1}$$ (if $i\ge 1$). In human genomes, the storage space with the maximized byte-encoded genotype array (MBEGs) will be reduced by nearly 11/12 or 15/16, compared to the common text format in VCF. Finally, BEG and MBEG can be pre-calculated and stored in an encoding table with $o(1)$ access time. The encoding table can be loaded in memory for fast encoding or decoding by direct mapping without computing. For instance, the encoding value of genotype $a\mid b$ is the element in row $a$ and column $b$ of the encoding table (Fig. 1b). The complete coding table of BEG and MBEG can be found in Additional file 1: Table S6.

#### Approximate minimum discrepancy ordering of variants (AMDO)

The diverse genotype of different variants in a block (especially for the non-conservative region) will result in dispersive genotype distributions (Fig. 2d), harming the genotype compression ratio. Thus, we propose an algorithm named approximate minimum discrepancy ordering (AMDO) to sort all variants within a block based on both the allele frequency and genotype distribution. AMDO provides an $o(nm)$ time complexity in the fast sorting process (compared to GTC with at least $o(n{m}^{2})$ time complexity), and significantly improves the compression ratio.

AMDO starts with extracting the genotype accumulated down-sampling features. Supposing that each block contains $M$ variants and $N$ subjects, a zero-count matrix is denoted as $C = {\left[{c}_{mn}\right]}_{M\times N}$, where ${c}_{mn}$ is the count of reference alleles (namely 0 alleles) of the ${m}^{\mathrm{th}}$ variant for the ${n}^{\mathrm{th}}$ subject. Then, the genotype vector of a variant $m$ ${C}_{m}=[{c}_{m0}, {c}_{m1}, \cdots , {c}_{m\left(N-1\right)}]$ is merged into a shorter $s$-element vector,

4 $$\begin{array}{c}{C}_{m}^{\left(l\right)}=\left[{C}_{m,0}^{\left(l\right)},{C}_{m,1}^{\left(l\right)},\cdots , {C}_{m,s-1}^{\left(l\right)}\right] \end{array}$$

where ${C}_{m,i}^{\left(l\right)}$ covers a maximum of *l* = ⎡*N/s*⎤ consecutive genotypes, and $s$ is 24 by default. Each element ${C}_{m,i}^{\left(l\right)}$ in the vector is defined as an accumulated count, i.e.:

5 $$\begin{array}{l}C_{m,i}^{\left(l\right)}=\sum\limits_{j=1.l}^{\min\;\left\{N\;-1,\;\left(i+1\right)l-1\right\}}\sum\limits_{k=i.l}^jc_{mk}=\left\{\begin{array}{ll}\sum\limits_{j=i\cdot l}^{\left(i+1\right)l-1}\left(\left(i+1\right)l-j\right)c_{mj}&,i< s-1\\ \sum\limits_{j=i\cdot l}^{N-1}\left(N-j\right)c_{mj}&,i=s-1\end{array}\right.\end{array}$$

The accumulation helps discriminate genotype distribution effectively. For example, according to (5), two zero-count vectors [0,1,1,2] and [2,1,1,0] have different accumulated counts, 7 and 9, respectively, although they have the same alternative allele counts.

All the variants in a block are divided into two groups, i.e., the biallelic and the multiallelic groups. In the biallelic variants group, the order of the variant ${v}_{i}$ and the biallelic variant ${v}_{j}$ is defined as the dictionary order of ${C}_{i}^{\left(l\right)}$ and ${C}_{j}^{\left(l\right)}$, which are described below:

- If $\exists {k}_{0}\in \left[0,s-1\right], \forall k\in \left[0,{k}_{0}-1\right]$, such that ${C}_{i, {k}_{0}}^{\left(l\right)} < {C}_{j, {k}_{0}}^{\left(l\right)}$, ${C}_{i, k}^{\left(l\right)}={C}_{j, k}^{\left(l\right)}$, then ${v}_{i}>{v}_{j}$;
- If $\exists {k}_{0}\in \left[0,s-1\right], \forall k\in \left[0,{k}_{0}-1\right]$, such that ${C}_{i, {k}_{0}}^{\left(l\right)}> {C}_{j, {k}_{0}}^{\left(l\right)}$, ${C}_{i, k}^{\left(l\right)}={C}_{j, k}^{\left(l\right)}$, then ${v}_{i}<{v}_{j}$;
- If $\forall k\in \left[0,s-1\right]$, such that ${C}_{i, k}^{\left(l\right)}={C}_{j, k}^{\left(l\right)}$, then ${v}_{i}={v}_{j}$.

On the contrary, the order will be inverted for multiallelic variants, which helps maximize the length of similar genotype vectors. Finally, the corresponding information of positions, alleles, and MBEGs for variants are sorted according to the ordered variants ($I=[{m}_{0}, {m}_{1}, \cdots , {m}_{M-1}]$).

### Merge data stream after compressing with advanced compressors

All the sorted MBEG and BEG codes in each block are further compressed by advanced compressors. Popular compression algorithms (e.g., Gzip, LZMA, and zlib) can achieve a compression ratio of 100 or more on genotypes. By default, we chose the ZSTD (short for Zstandard [20], https://github.com/facebook/zstd) because it provides the fastest speed with a similar compression ratio among the widely used compression algorithms. In detail, the byte codes of each variant are concatenated into a byte array ${B}_{1}$ directly. Next, the position of each variant is converted into 4 bytes, and then all variants’ positions are concatenated into a byte array ${B}_{2}$. Finally, the alleles of all variants are concatenated into another byte array ${B}_{3}$ with a “/” delimiter. Then, these concatenated data ${B}_{1}, {B}_{2}$ and ${B}_{3}$ are compressed by the latest ZSTD to produce ${\widehat{B}}_{1},{\widehat{B}}_{2}$ and ${\widehat{B}}_{3}$, respectively. The data entity is a long vector composed of three sections of compressed data, including encoded genotypes, positions, and alleles. Two types of information of each packed block, including abstract information and data entity, are subsequently written to the GTB file. The abstract information includes the chromosome number (1 byte), minimum and maximum positions (4 bytes each), number of biallelic variants (2 bytes each), number of multiallelic variants (2 bytes), length of ${\widehat{B}}_{1}$ (4 bytes), length of ${\widehat{B}}_{2}$ (3 bytes), length of ${\widehat{B}}_{3}$ (4 bytes), and magic code (1 byte) in a block. For other fields at the variant level (e.g., ID, INFO, Genotype Metrics), they are concatenated and compressed in the same way, then stored in the optional fields (Fig. 1a).

GBC can be integrated with different compression algorithms. ZSTD and LZMA algorithms have been embedded to compress each genotype block. We also reserve two types of compressors for developers to extend in the future.

### Memory control benefits to the efficiency of GBC on large-scale data

Saving memory is critical for large-scale projects, determining whether a user can easily compress the genotype data on ordinary desktops or servers. GBC overcomes the high memory load in processing large-scale datasets through three strategies. First, it has sufficient reusable buffers (also called context structures). Once the buffers are created, they can be re-used throughout the whole process of the task. Second, it adaptively adjusts the variant counts per block. The number of variants ($N$) contained in each block is set according to the subject size ($M$) automatically, as a block can store ${2}^{31}-2$ genotypes (approximately 2 GB size) at most. Third, it estimates memory usage during compression. The memory required is estimated based on the compression boundary estimation model (see details below).

Supposing a byte array of length $s$, whose original and estimated compressed sizes are ${R}_{s}$ and ${E}_{s}$ respectively. For small-scale data (length < 4 kB), the file’s head information (e.g., hash code, magic code, etc.) is larger than the genotype data in the compressed data. Thus, we set a fixed upper boundary $\beta$, which indicates the minimum memory required for compression. For large-scale data, the genotypes are the main part of the compressed data. Therefore, a good compression algorithm should ensure that the size of the compressed data will not exceed $\alpha (<\mathrm{inf })$ times of the original size. Here, the $\alpha$ is the main factor in the compression efficiency of large-scale data, which is estimated as:

6 $$\begin{array}{c}{E}_{s}=\mathrm{max}\left\{\widehat{\alpha }\cdot s,\widehat{\beta }\right\}\end{array}$$

In practice, we estimated $\alpha ,\beta$ by large-scale random data simulation experiments as follows because the real values are unknown:

7 $$\underset{\widehat{\alpha}, \widehat{\beta}}{\arg \min } \mathbb{I}\left(E_S< R_S\right)+\hat{\alpha}+\frac{\hat{\beta}}{10 \times 1024^2}$$

For large-scale data, $\beta$ can often be omitted. Thus, we defined $\widehat{\beta }=512, 1024, 1536,\cdots$, and the minimum $\widehat{\alpha }$ is obtained through grid search. By the estimation and calculation of our GBC, the compression boundary estimation model of ZSTD is ${E}_{s}^{\mathrm{ZSTD}}=\mathrm{max}\{1.0014\cdot s,7168\}$, while LZMA is ${E}_{\mathrm{s}}^{\mathrm{LZMA}}=\mathrm{max}\{1.0167\cdot s, 7680\}$, and Gzip is ${E}_{\mathrm{s}}^{\mathrm{Gzip}}=\mathrm{max}\{1.0031\cdot s,7680\}.$ Among the three algorithms, ZSTD takes the least amount of time in boundary estimation, indicating that ZSTD has a fairly fast compression speed, which enables GBC to rapidly create GTB files for downstream analyses.

#### Specify contig file to support non-human species genome compression

By default, GBC supports the genotype compression of human beings. It can also encode and compress genotypes of other haplotypic and diploid species. For non-human genomes, GBC only requires a different contig file(see Additional file 3: Note 3) to declare the label of the assigned chromosome (e.g., chrX, chrY, chrMT) and its ploidy. A contig file has “#chromosome,ploidy,length” as the header line, and then each line represents one chromosome. The option “--contig < file > ” is used as parameter input when compressing. Since only 1 byte is reserved for storing chromosome numbers in GTB format, we require that the number of chromosomes in the input contig file does not exceed 256.

#### Input multiple VCF files for compression

One or multiple VCF files can be merged into a single GTB file. For a single input file, GBC reads the file directly. For an input of multiple files, GBC first treats the file with the largest sample size as the major file and uses it to build the sample primary indexes. Other input files will be handled in turns after matching the sample indexes of the major file. In the matching process, the genotypes of missing subjects will be set as “.|.” subsequently to ensure that all the input files can be compressed together consistently in subsequent steps.

### Fast access and manage genotypes in the highly addressable GTB format

#### Parse GTB file and create an index table for fast access

When one accesses or manages a GTB file, all data except the block entity data is instantly loaded into the memory to construct a GTBManager. The GTBManager contains subject information, meta information, the basic status of the GTB (e.g., block size, compressor parameters), and the GTBTree (an index table for fast access, built from block abstract information). Throughout the program’s lifetime, the GTBManager of the read-in GTB files is always in the cache, which helps speed up multiple accesses to the data from the same files.

Here is the creation process of GTBTree. First, a GTBNode is built from the abstract information and the range of entity positions of a block (calculated by accumulating original lengths of compressed positions, alleles, and genotypes data). Then, all GTBNodes of the same chromosome are grouped. Finally, the GTBNodes of all chromosomes are collected to form the GTBTree. Because the compressed blocks may be out of order, we sort the GTBTree according to the minimum and maximum positions of each block’s chromosome. Then, the address of a variant is determined jointly by three indexes: the GTB file’s ID (if multiple GTB datasets were input), GTBNode, and the index within the block. Therefore, searching for a variant with a given position can be done by looking up the GTBNode(s) based on the boundary coordinates of the blocks at first. Then, the candidate GTBNode(s) can decompress the position data to verify its location within a block. Noticeably, the index table enables fast data access even if the original file is out of order.

#### The addressing algorithm to access location-specific genotypes

Accessing genotypes by column (i.e., subjects) is usually slower than by row (i.e., sites) because decompression involves all blocks. An addressing algorithm is developed to help quickly access a genotype from the MBEGs directly, which is implemented in two steps:

##### Step 1: Locating the start pointer of the ${m}^{\mathrm{th}}$ variant

In the MBEGs of a decompressed GTB block $i$, which contains ${b}_{i}^{\mathrm{biallelic}}$ biallelic variants and ${b}_{i}^{\mathrm{multiallelic}}$ multiallelic variants, the start pointer of the ${m}^{\mathrm{th}}$ variant in the MBEGs is calculated as:

8 $$\begin{array}{c}{P}_{m}=\left\{\begin{array}{ll}m\cdot \lceil\frac{N}{l}\rceil&,m<{b}_{i}^{\mathrm{biallelic}}\\ {b}_{i}^{\mathrm{biallelic}}\cdot \lceil\frac{N}{l}\rceil+\left(m-{b}_{i}^{\mathrm{biallelic}}\right)\cdot N &,else\end{array}\right.\end{array}$$

where $l$ is determined by the state of genotypes (phased: $l=3$; unphased: $l=4$), and $N$ is the number of subjects.

The address of the ${n}^{\mathrm{th}}$ subject in the multiallelic encoding sequence is $n$, whereas in the biallelic encoding sequence, which is retrieved by triples (index, groupIndex, codeIndex), where:

• index: The index of the ${n}^{\mathrm{th}}$ subject in the GTB subject information, that is $n$.
• groupIndex: The index of the MBEG code containing the genotype of ${n}^{\mathrm{th}}$ subject in the whole encoding sequence, calculated as ⎣*n/l*⎦.
• codeIndex: The index of the ${n}^{\mathrm{th}}$ subject in the MBEG code containing the genotype of ${n}^{\mathrm{th}}$ subject, calculated as $n \% l$.

##### Step 2: Decoding the genotype of the ${n}^{\mathrm{th}}$ subject

The biallelic genotype for the ${m}^{\mathrm{th}}$ variant of the ${n}^{\mathrm{th}}$ subject is stored in the $$\begin{array}{c}{\mathrm{codeIndex}}^{\mathrm{th}}\end{array}$$ BEG of the $${\left({P}_{m}+\begin{array}{c}\text{groupIndex}\end{array}\right)}^{\mathrm{th}}$$ $\mathrm{MBEG}$, and the multiallelic genotype for the ${m}^{\mathrm{th}}$ variant of the ${n}^{\mathrm{th}}$ subject is stored in the ${\left({P}_{m} + n\right)}^{\mathrm{th}}$ BEG.

#### The I/O optimized parallel framework for reading through GTB

While compression based on the GTB structure can facilitate parallel computing, the common bottleneck (often neglected) is disk I/O. Hard disks can have high bandwidth when reading or writing sequentially, but the addressing time (latency) is expensive. In many cases, multiple threads will not fasten the disk I/O and even make it worse (e.g., reading/writing a single 4-GB file to disk is much faster than multiple files with a total size of 4 GB). Based on the GTB structure, we used the producer/consumer model to coordinate the reading threads in GBC (see Additional file 2: Fig. S1a). The producer is responsible for mapping the requested variants onto GTB nodes and then adding involved GTB nodes into a task queue (a thread-safe collection in which multiple threads are added and data are updated concurrently). The consumers are the threads that read the tasks from the task queue, then load block entity data separately from the file and process the data (e.g., decompression). The model enables GBC to speed up analyses in two ways. First, the threads can be started before users process the file, which saves the time overhead of threads creating and recycling. Second, users can instantiate multiple consumers (i.e., number of threads) if the mapping speed of the producer is faster than the processing speed of the consumer(s). In addition, because the GTB structure packs genotypes of multiple variants into one block with remarkable compression ratios, it requires fewer I/O requests when retrieving multiple variants through multiple reading threads.

#### The cyclic locking model-based parallel algorithms to optimize decompression

Computing large-scale genotype data (such as decompression and LD calculation) often generates significant output data using multiple threads. Maintaining the order of the output data is a critical issue for massively parallel processing. The most common way to keep the order is using temporary files to store data and finally splice them into a single file. However, it usually takes up extra space and extra I/O costs, affecting the efficiency of parallel computing. Therefore, we propose an algorithm for parallel output and derive a theory for optimization.

The order problem in multiple threads is solved by a cyclic locking model (CLM, see Additional file 2: Fig. S1b and Fig. S1c) for decompression in which the current thread holds the lock of the next thread sequentially. When a reusable thread finishes decompression, it stays in memory to wait to write genotypes into disks until the previous thread has released its lock. The thread releases the lock of the next thread once it finishes writing the current data to disk. In detail, in a parallel decompression process with *t* threads, thread#0 holds the lock of thread#1, thread#1 holds the lock of thread#2, …, and thread#($t-1$) holds the lock of thread#0. Only the lock of thread#0 is released at the beginning of decompression. Any other thread cannot write to the disk because their previous threads do not release the lock. After thread#0 finishes the writing, it releases the lock of thread#1. The program continues this loop to decompress all the data. Therefore, CLM ensures that the decompressed data are output in the order of the GTB nodes.

It is known that the actual efficiency of parallelism usually depends on the device’s computing resources (such as memory, CPU cores, CPU clock speed, and I/O speed). Here, we estimate the theoretical processing time of parallel computation for CLM to derive an optimal number of threads. Assume that the source file (the file size is $S$) is split into $k$ tasks with approximately the same time overhead (the $k$ depends on the available memory). Denote the average output time of each task as ${t}_{\mathrm{o}}$, and the average processing time as ${t}_{\mathrm{p}}$. The time required to complete the task under *n* threads in parallel can be calculated as:

9 $$\begin{array}{c}t_{\mathrm{total}}^n=\lceil\frac kn\rceil\left(t_{\mathrm o}+t_{\mathrm p}\right)+\left(\left(k-1\right)\mod\;n\right)t_{\mathrm o}+\max\left\{\left(\left(n-1\right)t_{\mathrm o}-t_{\mathrm p}\right),0\right\}\left(\lceil\frac kn\rceil-1\right)+knt_\varepsilon\end{array}$$

where ${t}_{\varepsilon }$ is the time cost of thread switching (unavoidable); ${t}_{\mathrm{o}}$ and ${t}_{\mathrm{p}}$ depend on $k$ and the processing speed of the device. Thus, we can replace ${t}_{\mathrm{o}}=\frac{1}{{v}_{\mathrm{o}}}\cdot \frac{S}{k}$ and ${t}_{\mathrm{p}}=\frac{1}{{v}_{\mathrm{p}}}\cdot \frac{S}{k}$ in the above equation:

10 $$\begin{array}{c}t_{\mathrm{total}}^n=\lceil\frac kn\rceil\left(\frac1{v_{\mathrm o}}+\frac1{v_{\mathrm p}}\right)\frac Sk+\left(\left(k-1\right)\mod\;n\right)\frac S{kv_{\mathrm o}}+\max\left\{\left(\frac{\left(n-1\right)S}{{kv}_{\mathrm o}}-\frac S{{kv}_{\mathrm p}}\right),0\right\}\left(\lceil\frac kn\rceil-1\right)+knt_\varepsilon\end{array}$$

Here, ${v}_{o}$ and ${v}_{p}$ denotes the output speed and computation speed when processing unit files ($S/k$), and they can be estimated by the parameters of the benchmark device. Next, we use the overall efficiency of multi-threading to measure the benefits of multi-threading as:

11 $$\begin{array}{c}{E}_{n}=\frac{k\left({t}_{\mathrm{o}}+{t}_{\mathrm{p}}\right)}{{t}_{\mathrm{total}}^{n}}\end{array}$$

Generally, the parallelism will be efficient when $n\in N=\left\{n|n\le \lceil\frac{{t}_{\mathrm{p}}}{{t}_{\mathrm{o}}}\rceil+1\right\}$, while it is inefficient when $n>\mathrm{max}N$. Being inefficient means that thread switching and resource contention will increase time overhead. We showed multiple cases in Additional file 2: Fig. S1b and Fig. S1c and in Additional file 3 (I/O intensive task and computing-intensive task). The result indicated that the CLM algorithm is efficient for all parallel computing scenarios that require output data because $\mathrm{max}N\ge 2$ (see Additional file 2: Fig. S1d ~ Fig. S1g). Therefore, given the memory and CPU cores, the CLM theoretical estimation can help find the best file-splitting task number ($k$) and thread number $n$ on a device.

In addition, because the slow disk I/O speed will limit the ability of the parallel decompression algorithm, GBC also provides a way to directly decompress the data to BGZ format. Based on CLM, we have also developed a Java version of the parallel-bgzip compression algorithm, which has now been integrated into GBC as an auxiliary function.

#### Sort GTB by variants’ coordinates using a two-level index table

Large-scale genotype files (number of subjects and sites) are difficult to load into memory for directly sorting by variants’ coordinates. It is typical to use the temporary disk/memory space for sorting (e.g., BCFtools). In detail, the whole file is split into several chunks, and each chunk is sorted separately before being combined. However, BCFTools cannot sort very large datasets (e.g., VCF with 100,000 subjects and 1,000,000 variants) in 64 GB of memory. Thus, We propose a novel GTB-based sorting method to direct sort arbitrary scale genotype files within 4 GB of memory. The following is the sorting procedure on a chromosome (see Additional file 2: Fig. S2a):

*Step 1*: Construct a two-level index table of variants within the chromosome. The index table contains the positions and indexes of variants (chromosome position, GTBNode index, index within the block).

*Step 2*: Sort the index table by position.

*Step 3*: Move consecutive variants into a new GTB (balanced with the sample size) and sort the variants within the new block according to the GTBNode Index.

*Step 4*: The old blocks are decompressed sequentially by GBC, and the genotype data are expanded into BEGs by order of the variants in the new block.

*Step 5*: Perform compression (see the procedure in Fig. 2c~f) when all the genotypes of a new block have been added.

The purpose of sorting GTBNode Index (i.e., step 3) is to ensure that each old block will be decompressed at most once in the new block. The required memory is only equal to the size of each block because all threads share a single decompressor. We have shown that this algorithm makes the GBC 3 ~ 9 times faster (the “Results” section) than BCFtools under a single thread with no requirement for external disk space. Furthermore, the independence among the GTBs makes parallel sorting possible.

#### Merge multiple GTBs and identify inconsistent allele labels

Merging multiple genotype files is performed by recursively merging two files in a queue of length $L (L\ge 2)$. A file that is merged at the ${i}^{\mathrm{th}}$ time is decompressed and recompressed $L-i$ times. Thus, files containing more subjects should be placed at the back of the queue whenever possible. The minimum heap is used to optimize the merging process, and the node weights are the number of subjects in the file. The two files with the smallest weights in a heap are merged at first, and the merged file is added to the minimal heap. The process is repeated until only one file remains in the minimum heap (e.g., see Additional file 2: Fig. S2b).

The labeling of mismatched alleles can become a critical issue when merging genotypes from different batches. Therefore, GBC designs three functions to identify inconsistent allele labels:

- Check for allele frequency: the difference between the allele frequency of variant 1 and the allele frequency of variant 2 is less than the threshold (i.e., $\left|{\mathrm{AF}}_{1}-{\mathrm{AF}}_{2}\right|<0.1$). This will work for variants with minor allele frequencies much less than 0.5.
- Check for allele count: 2 × 2 column tables are constructed using the number of reference alleles at a variant of two batches to be merged. The chi-square tests are performed. If the hypothesis test rejects the ${H}_{0}$ hypothesis (i.e., the allele frequencies of the two variants are identical), then the variants in different batches cannot be considered potentially identical. Note that this will not be suitable for the scenario in that the two batches are used for cases and controls, respectively.
- Check for LD pattern: we first collect nearby variants of a given variant in which the absolute value of the genotypic correlation is over a threshold (say, 0.8) in two batches separately. Then, the positive signs of the correlation coefficients are counted in the two batches. If the numbers of signs are very different between the two batches, the allele labels should be flipped; otherwise, the allele labels are not flipped. This function can be used for variants with minor allele frequencies close to 0.5.

#### Evaluation and comparison

Three publicly available datasets were used for performance testing in this study, 1000GP3 [21], SG10K [14], and UKBB [3]. Besides, simulated datasets with various sample sizes (see Additional file 3: Note 2 and Additional file 1: Table S1) were also used to investigate the tools’ speed systematically. Note that the execution (compression, decompression, access) time is usually positively correlated with the scale of data. We excluded the non-genotype data in the VCF format to make a fair comparison. The compression ratio and speed of genotypes in phased and unphased were similar. So, in large-scale datasets like UKBB, we only tested each data set in one phasing state (phased or unphased).

We mainly compared GBC with PBWT [9], BGT [10], and GTC [12] because they were designed for a similar purpose to GBC—compressing genotypes into a fast-accessible format. GBC does not aim to exclusively achieve the highest compression ratio but an efficiently accessible compression. These tools have had different genotype formats, and “compression” refers to the conversion of genotypes to the formats designed by these tools. Another well-known tool, GQT [11], was excluded from the comparison tools due to its poor compression performance and inability to retrieve the specified variant and subject [12, 22]. Besides, we also excluded SeqArray [22] and GTRAC [23] from the list of comparison tools because SeqArray [22] is an R Library that does not provide standard command-line tools, and the output format of GTRAC [23] does not contain the standard VCF format. BCFTools [8], a popular method for fast accessing genotypes, is included in our comparison tool list. Although some latest studies argued that the access performance of BCFTools is poor [10–12], it offers almost the most comprehensive genotype management functions by far (which other tools do not have). Finally, GTShark [5] and Genozip [6], which provide a high compression ratio for genotype archiving, were also used to demonstrate the performance difference between fast-accessing and data-archiving methods. The versions and operating parameters of all software are shown in Additional file 3: Note 4.

The evaluation metrics for comparing basic compression performance include compression ratio, compression speed, and decompression speed with different sample sizes. For the comparison of accessing the compressed genotypes, the evaluation metrics included the speed of subject extraction and variant extraction (accessing random and contiguous variants, filtering by allele frequency). Then, for the performance of managing files, both GBC and BCFtools were used for compressed datasets, which avoided the interference of decompression and re-compression. Finally, GBC was compared with PLINK for the time overhead of population LD computation. Since both tools used bitwise operations, the time overhead of LD computation was linearly related to the sample size, which meaned that the performance in small-scale data can approximate the performance difference of LD computation in any sample scale.

### Additional information

#### System requirements

GBC was developed based on Oracle JDK 8. It is available on any computer device that supports or is compatible with Oracle JDK 8. Users are required to download and install the Oracle JDK or Open JDK firstly.

#### Computing environment

Experiments for UKBB datasets were run on the following configuration: 106 GB memory, Intel(R) Xeon(R) CPU X5560 @ 2.80 GHz 16 cores, and an SSD with sequential write speeds of up to 111 MB/s and sequential read speeds of up to 115 MB/s. All Other experiments were run on the following configuration: 32 GB 2933 MHz DDR4, Intel Core i7-10,700 2.9 GHz 8 cores, and a NvMe SSD with sequential write speeds of up to 1950 MB/s and sequential read speeds of up to 2400 MB/s.

## Supplementary Information

**Additional file 1: Table S1.** The basic compression performance comparison between GBC and alternative tools. **Table S2.** The comparison of GBC’s compression and decompression speed under multiple threads in the 1000GP3 dataset. **Table S3.** The data query performance comparison between GBC and alternative tools. **Table S4.** The comparison of LD calculation speed between GBC and alternative tools in the 1000GP3 and SG10K datasets. **Table S5.** The file management performance comparison between GBC and alternative tools. **Table S6.** BEG and MBEG coding tables for genotypes of diploid species.

**Additional file 2: Fig. S1.** Using CLM algorithm to achieve ordered output during parallel computation of large-scale data. **Fig. S2.** Optimized file management (sorting and merging) based on GTB.

**Additional file 3: Note 1.** A detailed description of GTB file. **Note 2.** The generation method of simulation genotypes. **Note 3.** The format of the contig file. **Note 4.** Examined programs.

**Additional file 4.** Review history.

## Data Availability

In this study, partial genotype data from the UK Biobank was accessed through a collaboration with applications no.86920. Data are available for bona fide researchers upon application to the UK Biobank, and the high-coverage whole-genome sequencing data of SG10K is available on https://ega-archive.org with accession number EGAS00001003875 (14). The 1000GP3 dataset (22) in this paper is shown in https://pmglab.top/genotypes (dataset is also available on the FTP site at http://ftp.1000genomes.ebi.ac.uk/vol1/ftp/release/20130502/), which do not require access rights. Lastly, all simulation data used in this study were generated using the method described in Additional file 3: Note 2, and this method has been implemented in the VCFGenerator.java, which is available at https://doi.org/10.5281/zenodo.7737556.
