# Supplementary material for: GBC: a parallel toolkit based on highly addressable byte-encoding blocks for extremely large-scale genotypes of species
Source: Genome Biol. 2023 Apr 17;24:76. doi: 10.1186/s13059-023-02906-z (PMC10108510; doi:10.1186/s13059-023-02906-z)
Supplement: Supplementary file 2 — Additional file 2: Fig. S1. Using CLM algorithm to achieve ordered output during parallel computation of large-scale data. Fig. S2. Optimized file management (sorting and merging) based on GTB. [file 13059_2023_2906_MOESM2_ESM.docx]

**Supplementary Figure 1**


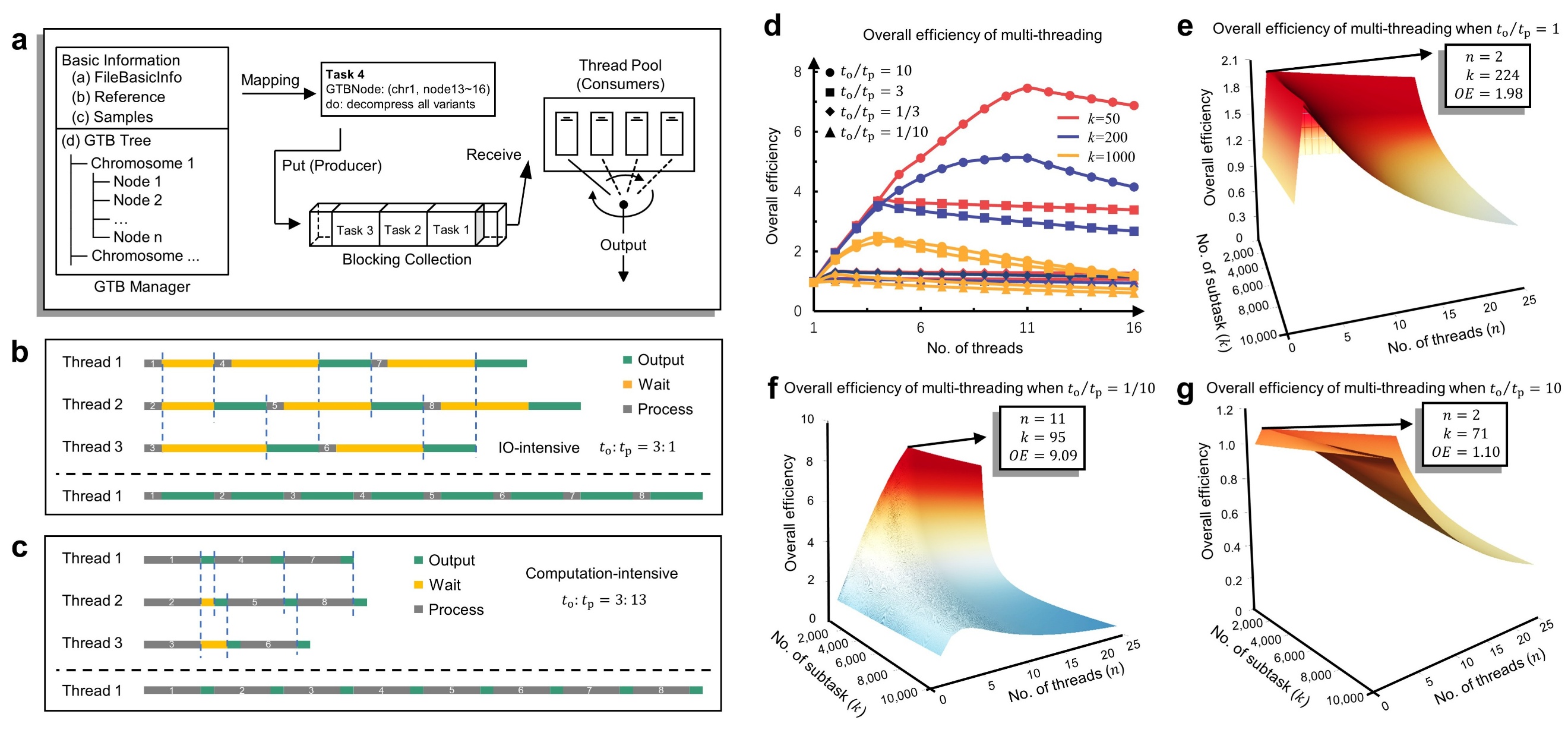


**Fig.S1 |** **Using CLM algorithm to achieve ordered output during parallel computation of large-scale data.** **a**, Using the producer/consumer model and CLM for multi-threaded architecture design with GTB. **b**, Analytical plot of the time overhead when parallelizing the output using the CLM algorithm at $t_{o}:t_{p}=3:1$. **c**, Analytical plot of the time overhead when parallelizing the output using the CLM algorithm at $t_{o}:t_{p}=3:13$. **d**, Overall efficiency of multi-threading under different threads $n$, time cost ratio between $t_{o}$ and $t_{p}$, number of subtasks $k$. **e**, Overall efficiency of multi-threading under $t_{o}:t_{p}=1:1$. **f**, Overall efficiency of multi-threading under $t_{o}:t_{p}=1:10$. **g**, Overall efficiency of multi-threading under $t_{o}:t_{p}=10:1$.

**Supplementary Figure 2**


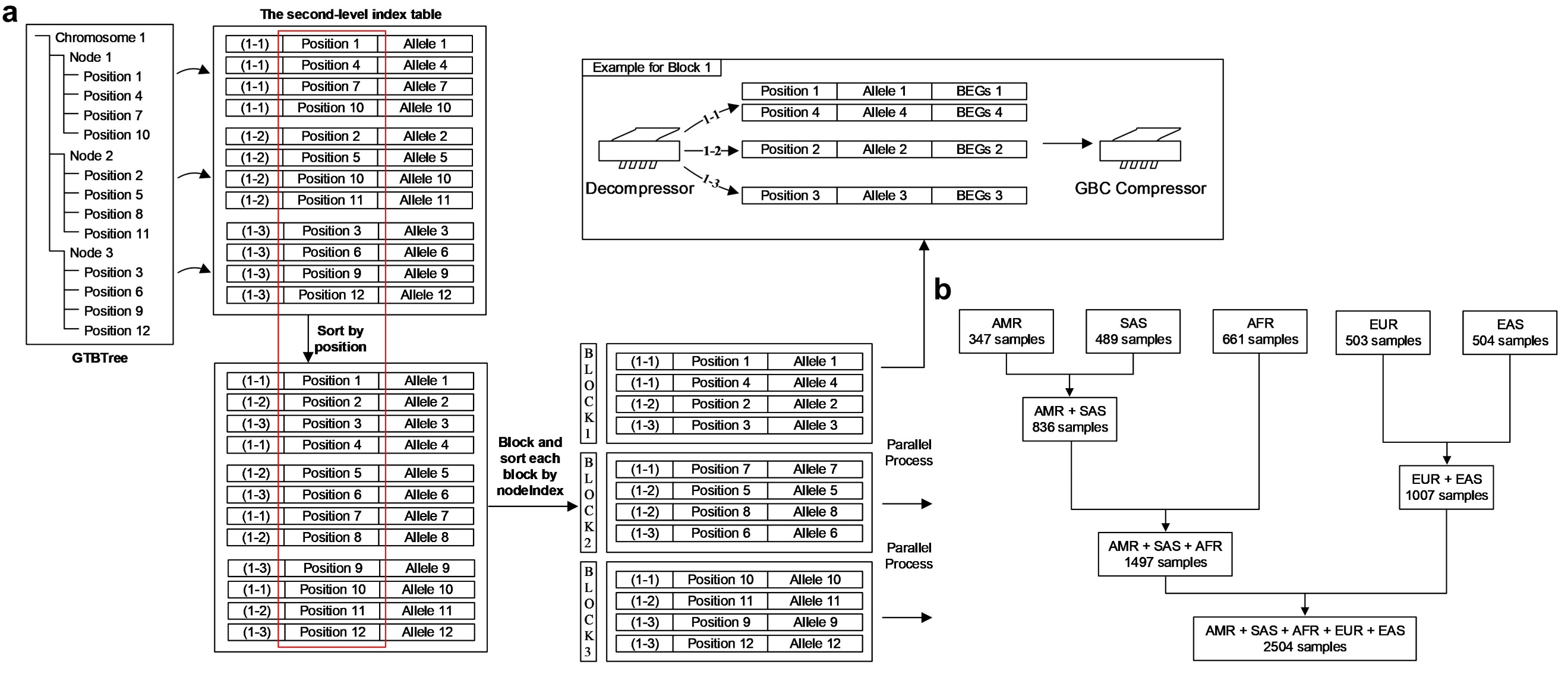


**Fig.S2 |** **Optimized file management (sorting and merging) based on GTB. a**, Sort GTB by variant’s coordinates using a two-level index table. **b**, The merging process of multiple files is streamlined using a minimal heap, illustrated here with the practical example of 1000GP3.
