# Supplementary material for: GBC: a parallel toolkit based on highly addressable byte-encoding blocks for extremely large-scale genotypes of species
Source: Genome Biol. 2023 Apr 17;24:76. doi: 10.1186/s13059-023-02906-z (PMC10108510; doi:10.1186/s13059-023-02906-z)
Supplement: Supplementary file 3 — Additional file 3: Note 1. A detailed description of GTB file. Note 2. The generation method of simulation genotypes. Note 3. The format of the contig file. Note 4. Examined programs. [file 13059_2023_2906_MOESM3_ESM.docx]

**Supplementary Notes**

| **GBC: A parallel toolkit based on highly addressable byte-encoding blocks for extremely large-scale genotypes of species** |
| --- |

**Liubin Zhang, Yangyang Yuan, Wenjie Peng, Bin Tang, Qiang Wang, Mulin Jun Li, Hongsheng Gui, Miaoxin Li^†^**

**TABLE OF CONTENTS**

[Supplementary Note 1. A detailed description of GTB file 1](#_Toc129951845)

[Supplementary Note 2. The generation method of simulation genotypes 3](#_Toc129951846)

[Supplementary Note 3. The format of the contig file 4](#_Toc129951847)

[Supplementary Note 4. Examined programs 4](#_Toc129951848)

# Supplementary Note 1. A detailed description of GTB file

The structure of the GTB file is shown in Fig.S1. Here, we describe the meaning of each field and its generation process.

1) Magic Code (2 bytes)

The first two bytes store the basic information of the file, including:

- Estimate Block Size: 4bits, the estimated disk space occupied by each block after being decompressed to VCF format. The mapping between Magic Code and disk space occupied is shown in Table 1.
- Ordered GTB: 1bit, whether the GTB file is in order. After compression, GBC will scan all abstract information and judge whether each block's range of position (Minimum Position, Maximum Position) is overlapping. The existing overlapping nodes indicate an unordered file, thus the mark information is 0, otherwise, it is 1.
- Compressor: 2 bits, the core compressor used in compressing. By default, 0 stands for ZSTD and 1 stands for LZMA. Besides, 2 and 3 are reserved and can be extended by other developers. The compressor is defined in the compressor/ICompressor.java file, and users can make extensions according to the LZMA.
- Phased: 1 bit, the state of phased or unphased genotypes. By default, 0 stands for the unphased file, and value '1' stands for the phased file.
- Block Size Type: 3 bits, the type of block size. Parameter values range from 0 to 7. Reference information for each block size type is shown in Table 2.
- Compression Level: 5 bits, the compression level to use when the compressor work. In the current implementation, ZSTD algorithm supports the range of [0,22], and LZMA supports the range of [0,9].

**Table 1. Representation of estimate block size by bit-encoding**

| Magic Code | Means | Magic Code | Means |
| --- | --- | --- | --- |
| 0000 | Broken GTB | 1000 | 128 MB < size <= 192 MB |
| 0001 | size <= 2 MB | 1001 | 192 MB < size <= 256 MB |
| 0010 | 2 MB < size <= 4 MB | 1010 | 256 MB < size <= 384 MB |
| 0011 | 4 MB < size <= 8 MB | 1011 | 384 MB < size <= 512 MB |
| 0100 | 8 MB < size <= 16 MB | 1100 | 640 MB < size <= 768 MB |
| 0101 | 16 MB < size <= 32 MB | 1101 | 768 MB < size <= 896 MB |
| 0110 | 32 MB < size <= 64 MB | 1110 | 896 MB < size <= 1 GB |
| 0111 | 64 MB < size <= 128 MB | 1111 | > 1 GB (Suggest to bgzf) |

**Table 2. Representation of block size type by bit-encoding**

| Block Size Type | Block Size | Sample Size | Block Size Type | Block Size | Sample Size |
| --- | --- | --- | --- | --- | --- |
| 111 | 16384 | $\leq131071$ | 011 | 1024 | $\leq2097151$ |
| 110 | 8192 | $\leq262143$ | 010 | 512 | $\leq4194303$ |
| 101 | 4096 | $\leq524287$ | 001 | 256 | $\leq8388607$ |
| 100 | 2048 | $\leq1048575$ | 000 | 128 | $\leq16777215$ |

2) Block Numbers

After decompressing all the data, the number of Block Abstract Information will be stored in 3 bytes. Actually, a single GTB file can store 16,777,215 blocks, and each block can store more than 128 variants. Thus, a single GTB file can store at least 2,147,483,520 variants, which is enough for the whole human genome.

3) Meta Information

The meta information "##<key>=<value>" fields of the input file are saved. For multiple file inputs, GBC will save the information of the first file. It will be empty if there is no such information in the input files.

4) Subject Information

The compressed subjects come from the input files. In large projects, sample names often have similar prefixes, and compression may help save space. The Subject Information includes the length of the compressed data (4 bytes) and the compressed data.

5) Block Entity Data

The compressed data is stored in this area. Genotype data, position data and allele data generated from the same block will be continuously written in this area, and the corresponding compressed size is recorded in the Block Abstract Information to help locate each block entity data and the pointer range of each part.

6) Abstract Block Information

The chromosome number is the same within each block. Each block of the Block Abstract Information, the initial 1 byte represents the chromosome. Then, 8 bytes were used to describe the minimum and maximum position of each block, which is the basis for rapid variants localization. Next, the numbers of biallelic variants and multiallelic variants are recorded. Then, 11 bytes are used to represent the length of compressed genotype data, compressed position data and compressed allele data in Block Entity Data area. The last Magic Code is the larger of the original (decompressed) length of MBEGs and Alleles in each block (Table 3).

**Table 3. Representation of estimated maximum size of MBEGs and Alleles by bit-encoding**

| Magic Code | Means | Magic Code | Means |
| --- | --- | --- | --- |
| 0000 | 0 < size <= 2 MB | 1000 | 192 MB < size <= 256 MB |
| 0001 | 2 MB < size <= 4 MB | 1001 | 256 MB < size <= 384 MB |
| 0010 | 4 MB < size <= 8 MB | 1010 | 384 MB < size <= 512 MB |
| 0011 | 8 MB < size <= 16 MB | 1011 | 640 MB < size <= 768 MB |
| 0100 | 16 MB < size <= 32 MB | 1100 | 768 MB < size <= 896 MB |
| 0101 | 32 MB < size <= 64 MB | 1101 | 896 MB < size <= 1 GB |
| 0110 | 64 MB < size <= 128 MB | 1110 | 1 GB < size <= 1.5 GB |
| 0111 | 128 MB < size <= 192 MB | 1111 | 1.5 GB < size <= 2GB - 2 B |

# Supplementary Note 2. The generation method of simulation genotypes

To evaluate the speed (including compression, decompression and data query) of GBC and other alternative methods on various scale datasets, datasets spanning multiple orders of magnitude are necessary. However, limited by the amount of the real data, and the cost of generating real data is too high, thus, the most straightforward strategy is to sample from the existing large datasets or conduct simulation. Here, we adopted simulation to avoid data bias and sampling bias. The simulated data includes the positions, alleles and genotypes. It should be noted that these datasets are randomly generated according to the allele frequency, and there is no linkage. Therefore, the simulation datasets only make sense on the speed comparison. Below is the detailed process for generating data of positions, alleles and genotypes.

1) Positions:

- For an ordered genome file, we generate positions as follows:

$$Position_{i}= 10 \left( i+1 \right)+p_{i}^{\left( 1 \right)}, p_{i}^{\left( 1 \right)}\sim U(1, 9)$$

Where $i$ is the index of a position (start from 0); $p_{i}^{\left( 1 \right)}$ is a uniformly distributed random integer over the interval [1, 9].

- For an unordered genome file, we generate positions as follows:

$$Position_{i}=start + p_{i}^{\left( 2 \right)}, p_{i}^{\left( 2 \right)}\sim U(0, 2^{29})$$

Where $start\geq1$, which represents the minimum position for these variants.

2) Alleles:

Since alleles are not our primary concern, we use a simple method for a generation. Alleles are randomly sampled from A, T, C, and G non-repetitively with the first base as REF and the second base as ALT.

3) Genotypes:

Set $p^{\left( 3 \right)}$ as the missing rate of genotypes, $p^{\left( 4 \right)}$ is the probability of major allele being ALT (0), $p^{\left( 5 \right)}$ as the probability of major allele in each haplotype allele. All the variants are bi-allelic sites. And the genotypes of a variant is generated as follows:

| Algorithm: The generation method of simulation genotypes |
| --- |
| Input $p^{\left( 3 \right)}$, $p^{\left( 4 \right)}$, $p^{\left( 5 \right)}$ |
| Process  if (random.nextFloat() < $p^{\left( 4 \right)}$) then  let majorAllele = 0, minorAllele = 1  else  let majorAllele = 1, minorAllele = 0  for *j* in [0, sampleSize)  if (random.nextFloat() < $p^{\left( 3 \right)}$) then  genotype*_j_* = “.\|.”  else  if (random.nextFloat() < $p^{\left( 5 \right)}$) then  genotype*_j_* = majorAllele + “\|”  else  genotype*_j_* = minorAllele + “\|”  end if    if (random.nextFloat() < $p^{\left( 5 \right)}$) then  genotype*_j_* = genotype*_j_* + majorAllele  else  genotype*_j_* = genotype*_j_* + minorAllele  end if  end if  end for |
| Output Genotype sequence of variant $i$ |

# Supplementary Note 3. The format of the contig file

GBC uses the following file as the default contig file. The chromosome labels 'X' and 'chrX' are identified as chromosome id '23' in this file. The user can construct similar files using the argument 'java -jar gbc.jar index $inputFile$' or 'java -jar gbc.jar index $inputFile$ --deep-scan' to adapt the genome compression for different species.

| ##reference=https://www.ncbi.nlm.nih.gov/grc/human/data?asm=GRCh38.p13  #chromosome,ploidy,length  1,2,248956422  2,2,242193529  3,2,198295559  4,2,190214555  5,2,181538259  6,2,170805979  7,2,159345973  8,2,145138636  9,2,138394717  10,2,133797422  11,2,135086622  12,2,133275309  13,2,114364328  14,2,107043718  15,2,101991189  16,2,90338345  17,2,83257441  18,2,80373285  19,2,58617616  20,2,64444167  21,2,46709983  22,2,50818468  X,2,156040895  Y,1,57227415  MT,2,4485509 |
| --- |

# Supplementary Note 4. Examined programs

This section focuses on the running commands of the tools involved in the experiments in the paper. The contents wrapped in a pair of US-dollar symbols (i.e., $...$) are the user's parameters to modify. For example, $inputFile$ needs to be replaced with the correct vcf or vcf.gz path. Some tools don't support exporting to vcf.gz format. For uniform comparison, tools other than GBC use ‘| bgzip -c -l 6 > $outputFile$’ to export to vcf.gz format. The runtime of all tools except GBC is obtained through the /usr/bin/time directive obtains the exact runtime, while GBC through the built-in 'System.currentTimeMillis()' to exclude the interference of JVM startup time. In the experiments, all instructions were run 5 times, and the best result was taken as the final result.

## PBWT (Version: 3.0-8c25e5c, https://github.com/richarddurbin/pbwt)

- compression: ./pbwt -readVcfGT $inputFile$ -writeAll $outputFile$
- decompression: ./pbwt -readAll $inputFile$ -writeVcf - | > $outputFile$
- sample query: ./pbwt -readAll $inputFile$ -selectSamples $sampleFile$ -writeVcf - | > $outputFile$
- range of continuous variants query: ./pbwt -readAll $inputFile$ -subrange $start$ $end$ -writeVcf - | > $outputFile$

## BGT (Version: 1.0-r284-dirty, https://github.com/lh3/bgt)

- compression: ./bgt import -S -o $outputFile$ $inputFile$
- decompression: ./bgt view $inputFile$ > $outputFile$
- sample query: ./bgt view -s ,$sample1,sample2,...$ $inputFile$ > $outputFile$ or ./bgt view -s $ sampleFile$ $inputFile$ > $outputFile$
- range of continuous variants query: ./bgt view -r $chrom$:$start$-$end$ $inputFile$ > $outputFile$
- range of allele frequency query: ./bgt view -f 'AF>=$minAF$&&AF<=$maxAF$' $inputFile$ > $outputFile$

## GTC (Version: v1.1, https://github.com/refresh-bio/GTC)

- compression: ./gtc compress -o $outputFile$ $inputFile$
- decompression: ./gtc view -C -o $outputFile$ $inputFile$
- sample query: ./gtc view -C -s $sample1,sample2,...$ -o $outputFile$ $inputFile$
- range of continuous variants query: ./gtc view -C -r $chrom$:$start$-$end$ -o $outputFile$ $inputFile$
- range of allele frequency query: ./gtc view -C -minAF $minAF$ -maxAF $maxAF$ -o $outputFile$ $inputFile$

## BCFtools (Version: 1.12, https://github.com/samtools/bcftools)

- build index for ordered vcf.gz: ./bcftools index $inputFile$
- sample query: ./bcftools view -S $sampleFile$ $inputFile$ -o $outputFile$
- range of continuous variants query: ./bcftools view -r $chrom$:$start$-$end$ $inputFile$ -o $outputFile$
- random variants query: ./bcftools view -R $positionFile$ $inputFile$ -o $outputFile$
- range of allele frequency query: ./bcftools view --min-af $minAF$ --max-af $maxAF$ $inputFile$ -o $outputFile$
- split vcf.gz by chromosome: ./bcftools view -r $chromosome$ $inputFile$ -O z -o $outputFile$
- concatenate genotypes with identical subjects: ./bcftools concat $inputFile1$ $inputFile2$... -o $outputFile$
- merge genotypes with non-overlapping samples: ./bcftools merge $inputFile1$ $inputFile2$... -o $outputFile$
- sort vcf.gz by coordinate: ./bcftools sort $inputFile$ -o $outputFile$

## VCFtools (Version: 0.1.16, https://github.com/vcftools/vcftools)

- calculate LD score using $D^{'}$ and $r^{2}$ method: ./vcftools --gzvcf $inputFileName$ --ld-window-bp 10000 --hap-r2 --out $outputFileName$ --min-r2 0.2 --maf 0.05
- calculate LD score using Pearson $r^{2}$ method: ./vcftools --gzvcf $inputFileName$ --ld-window-bp 10000 --geno-r2 --out $outputFileName$ --min-r2 0.2 --maf 0.05

## PLINK (Version: v1.90b6.24, https://www.cog-genomics.org/plink/)

- calculate LD score using $D^{'}$ and $r^{2}$ method: ./plink --vcf $inputFile$ --ld-window 10000 --ld-window-kb 10 --ld-window-r2 0.2 --out $outputFile$ --maf 0.05 --r2 dprime --threads $threadNum$
- calculate LD score using Pearson $r^{2}$ method: ./plink --vcf $inputFile$ --ld-window 10000 --ld-window-kb 10 --ld-window-r2 0.2 --out $outputFile$ --maf 0.05 --r2 --threads $threadNum$

## GTShark (Version: 1.1, https://github.com/refresh-bio/GTShark)

- compression: ./gtshark compress-db $inputFile$ $output$.gtshark
- decompression: ./gtshark decompress-db $inputFile$ $output$
- query a single sample (output as VCF format): ./gtshark extract-sample $inputFile$ $sample$ $outputFile$

## Genozip (Version: 14.0.12, https://www.genozip.com)

- compression: genozip $inputFile$ -o $outputFile$ --best --input vcf --force --no-test --threads $threads$
- decompression: genocat $inputFile$ -f -o $outputFile$ -z 6 -@ 1
- random variants query: genocat $inputFile$ -f -o $outputFile$ -z 6 -@ 1 --regions-file $positionFile$
- range of continuous variants query: genocat $inputFile$ -f -o $outputFile$ -z 6 -@ 1 -r $chrom$:$start$-$end$
- sample query: genocat $inputFile$ -f -o $outputFile$ -z 6 -@ 1 -s $sample1,sample2,...$

## GBC (Version: 1.2, <https://doi.org/10.5281/zenodo.7737556>, Online Manual: https://pmglab.top/gbc/history/v1.2)

About: Version 1.x is the version described and tested in this publication, which supports all features and has the best access performance at the genotype level. **The latest version of GBC is released at http://pmglab.top/gbc.**

In the following commands, ‘GBC’ is short for the command ‘java -Xms4g -Xmx4g -jar gbc.jar’.

- build contig file: GBC index $inputFile$ or GBC index $inputFile$ --deep-scan
- compression: GBC build $inputFile$ -o $outputFile$ -t $threads$
- decompression (to vcf format): GBC extract $inputFile$ -o $outputFile$ --o-text -t $threads$
- decompression (to vcf.gz format): GBC extract $inputFile$ -o $outputFile$ --o-bgz -t $threads$
- sample query: GBC extract $inputFile$ -o $outputFile$ --subject $sample1,sample2,...$ or GBC extract $inputFile$ -o $outputFile$ --subject @$sampleFile$ -t $threads$
- range of continuous variants query: GBC extract $inputFile$ -o $outputFile$ --range $chrom$:$start$-$end$ -t $threads$
- random variants query: GBC extract $inputFile$ --output $outputFile$ --random $positionFile$ -t $threads$
- range of allele frequency query: GBC extract $inputFile$ --output $outputFile$ --seq-af $minAF$-$maxAF$ -t $threads$
- split GTB by chromosome: GBC edit $GTBFile$ -o $outputDir$ --split
- concatenate genotypes with identical subjects: GBC edit $inputFile$ --concat $GTBFile1$ $GTBFile2$... -o $outputFile$
- merge genotypes with non-overlapping samples: GBC merge $GTBFile1$ $GTBFile2$... -o $outputFile$ -t $threads$
- sort GTB by coordinate: GBC rebuild $inputFile$ -o $outputFile$ -t $threads$
- calculate LD score using $D^{'}$ and $r^{2}$ method: GBC ld $inputFile$ --ld-window-bp 10000 --min-r2 0.2 --maf 0.05 --model --hap -o $outputFile$ -t $thread$
- calculate LD score using Pearson $r^{2}$ method: GBC ld $inputFile$ --ld-window-bp 10000 --min-r2 0.2 --maf 0.05 --model --geno -o $outputFile$ -t $thread$
