# Supplementary material for: GBC: a parallel toolkit based on highly addressable byte-encoding blocks for extremely large-scale genotypes of species
Source: Genome Biol. 2023 Apr 17;24:76. doi: 10.1186/s13059-023-02906-z (PMC10108510; doi:10.1186/s13059-023-02906-z)
Supplement: Supplementary file 4 — Additional file 4. Review history. [file 13059_2023_2906_MOESM4_ESM.docx]

Review History

**First round of review**

**Reviewer 1**

**Were you able to assess all statistics in the manuscript, including the appropriateness of statistical tests used?** Yes: no statistical test was provided

**Were you able to directly test the methods?** Yes

**Comments to author:**

The authors present a new tool, GBC, to effectively deal with large-scale genotypes.

- The methods are appropriate and well described.

- the conclusions are adequately supported by the data shown.

- Sufficient details are provided to allow replication and comparison with related analyses that may have been performed

- The method is of interest to the community dealing with analyses of genomic variants, as it can significantly speed up the computations typically carried out in these data.

Some comments:

- Missing some relevant references, such as the previously proposed method GTRAC (https://academic.oup.com/bioinformatics/article/32/17/i479/2450767), and probably the effort from GA4GH on developing new standards as well.

- Quality of figures should be improved (vectorized them for example).

- Fig.2a, relative advantage ratio is not defined.

- Fig. 2a, caption says several files were tested, but it is not specified then how the metrics are computed. Do those refer to the average across all files?

- Typo page 9 line 58, it should be "replace" instead of "relace"

- In conclusions: population PCA not defined.

- In conclusions, I miss more discussion on the future possible extension to include other fields of the VCF file. In the supplementary methods section "1.1. the structure of GTB" it says that other fields can be concatenated and store. I think this can be important if someone wants to store additional information.

- QC seems to be applied by default. I am wondering whether this option can be desactivated.

**Reviewer 2**

**Were you able to assess all statistics in the manuscript, including the appropriateness of statistical tests used?** Yes: No further statistical tests are required

**Were you able to directly test the methods?** Yes

**Comments to author:**

In this work the authors present Genotype Blocking Compressor (GBC), a method for rapidly compressing large-scale genotypes into a fast-accessible and highly parallelizable format.

While the paper is well written and the method shows improvement over some previously proposed methods, the work has several major and critical shortcomings:

The authors benchmark their propose method GBC against GTC, BGT, PWBT, bcfTools. However they have missed recent key work on both VCF and Genotype compression such as:

GTShark (https://doi.org/10.1093/bioinformatics/btz508),

VCFShark (https://doi.org/10.1093/bioinformatics/btab211),

GenoZip (https://doi.org/10.1093/bioinformatics/btab102),

spVCF (https://doi.org/10.1093/bioinformatics/btaa1004).

Without a fair comparison against the above methods, it is not possible to make a fair assessment of the relevance of the proposed method.

In addition, the authors propose the compression of Genotypes rather than the whole VCF file. Note that while genotypes (and allele information) are the key information on VCF files, these files contained other information that should not be missed, such as quality metrics and annotations, among others. In order for the proposed method to see some real adoption, it is of utmost importance to include other components of the VCF file in the compressed file. Further, the proposed method should ideally be able to access these components efficiently (for example to help users efficiently filter the VCF files based on different quality metrics).

minor comments:

Please do not use Chinese in the English version of the software website.

**Authors Response**

**Point-by-point responses to the reviewers’ comments:**

**Reviewer #1:**

The authors present a new tool, GBC, to effectively deal with large-scale genotypes.

- The methods are appropriate and well described.

- the conclusions are adequately supported by the data shown.

- Sufficient details are provided to allow replication and comparison with related analyses that may have been performed

- The method is of interest to the community dealing with analyses of genomic variants, as it can significantly speed up the computations typically carried out in these data.

Some comments:

**Q1:** Missing some relevant references, such as the previously proposed method GTRAC (https://academic.oup.com/bioinformatics/article/32/17/i479/2450767), and probably the effort from GA4GH on developing new standards as well.

**Response:** We thank the reviewer for this comment. We added the relevant references for SeqArray and GTRAC on Page 21 Line 574~576 of the revised manuscript. Besides, a recent study GTC (https://doi.org/10.1093/bioinformatics/bty023) indicated that both SeqArray and GTRAC performed worse than GTC, and our results showed that GBC outperformed GTC in larger-scale genotype datasets.

As for the GA4GH, we create an interface conversion class for GTB read objects to convert GTB-API to GA4GH-API (<https://ga4gh-schemas.readthedocs.io/en/latest/>). This will help other tools compatible with the GA4GH specification to apply the GTB format and GBC tools with low development costs. We added the relevant reference for GA4GH on Page 3 Line 60.

**Q2:** Quality of figures should be improved (vectorized them for example).

**Response:** We are sorry about this problem. The final PDF automatically generated by the web server was compressed and blurred our images. In the revised manuscript, we adjusted the digital dpi to 300, and also supplemented all images with high-quality in supporting materials.

**Q3:** Fig.2a, relative advantage ratio is not defined. Fig. 2a, caption says several files were tested, but it is not specified then how the metrics are computed. Do those refer to the average across all files?

**Response:** We thank the reviewer for this comment and apologize for this vague statement. In this new version, we changed the "relative advantage ratio" to "standardized performance ratio", and described how we did on Page 7 Line 186~188. Briefly, the testing results of each tool were divided by the datasets' sample size and variant size first. The results for all datasets were then averaged for each tool. The "standardized performance ratio" was finally obtained by scaling the best-averaged result to 1 and all other results to less than 1.

**Q4:** Typo page 9 line 58, it should be "replace" instead of "relace"

**Response:** Thanks. We have corrected this typo in the new version and checked for other possible errors throughout our whole manuscript again.

**Q5:** In conclusions: population PCA not defined.

**Response:** Sorry for not defining this term. We define it in the revised manuscript. It is "Principal Component Analysis (PCA) of genotypes to detect population stratification". We also added a reference of population PCA on Page 10 Line 256.

**Q6:** In conclusions, I miss more discussion on the future possible extension to include other fields of the VCF file. In the supplementary methods section "1.1. the structure of GTB" it says that other fields can be concatenated and store. I think this can be important if someone wants to store additional information.

**Response:** We thank the reviewer for the constructive comments. In this new version, we improved the structure of GTB to store additional information from VCF (Page 11, Fig. S1a). GBC only stores genotypes by default, and all other fields of interest in VCF(e.g., all Meta, ID and INFO of variants, genotype quality) now can be included in compression via the new option `--addFields`. Below are the Code Examples with `--addFields`(for input) and `--selectFields`(for output).

**Q7:** QC seems to be applied by default. I am wondering whether this option can be desactivated.

**Response:** We thank the reviewer for this comment. To ensure the quality of variants and genotypes for follow-up analysis, QC is applied by default by GBC. However, users can also add the parameter '--no-qc' to deactivate the quality control when building GTB files.

| **GBC-2.0** | **http://pmglab.top/gbc/download/gbc-2.0.zip** |
| --- | --- |
| **Example** | **rare.disease.hg19.vcf.gz download from http://pmglab.top/gbc/download/example.zip** |
| # build GTB file, store INFO field and genotype metrics java -jar gbc-2.0.jar build ./example/rare.disease.hg19.vcf.gz \ --addFields GENOTYPE,INFO,META,GENOTYPE_METRIC --no-qc  # The following commands help the user to select which components in the VCF file to export and filter variants or genotypes by these fields: java -jar gbc-2.0.jar view ./example/rare.disease.hg19.gtb \ --selectFields GENOTYPE,GENOTYPE_METRIC \ --gty-gq 20 --gty-dp 4 --gty-ad 10 --gty-pl 20 --seq-qual 30 --seq-fs 60 --seq-mq 20 | |
| **Example** | **clinvar_20211225.vcf.gz download from**  **https://ftp.ncbi.nih.gov/pub/clinvar/vcf_GRCh37/archive_2.0/2022/clinvar_20211225.vcf.gz** |
| # build GTB file  java -jar gbc-2.0.jar build ./example/clinvar_20211225.vcf.gz \ --addFields INFO,META --no-qc  # extract data to the specified file  java -jar gbc-2.0.jar view ./example/clinvar_20211225.gtb ./example/clinvar_20211225.vcf | |

**Reviewer #2:**

In this work the authors present Genotype Blocking Compressor (GBC), a method for rapidly compressing large-scale genotypes into a fast-accessible and highly parallelizable format.

While the paper is well written and the method shows improvement over some previously proposed methods, the work has several major and critical shortcomings:

**Q1:** The authors benchmark their propose method GBC against GTC, BGT, PWBT, bcfTools. However they have missed recent key work on both VCF and Genotype compression such as:

GTShark (https://doi.org/10.1093/bioinformatics/btz508),

VCFShark (https://doi.org/10.1093/bioinformatics/btab211),

GenoZip (https://doi.org/10.1093/bioinformatics/btab102),

spVCF (https://doi.org/10.1093/bioinformatics/btaa1004).

Without a fair comparison against the above methods, it is not possible to make a fair assessment of the relevance of the proposed method.

**Response:** We thank the reviewer for this comment. In this new version, we compared the compression performance of these methods using a UKBB exon genotype dataset of 469,835 subjects. The results (GBC: 3.27GB, GTShark: 2.76GB, VCFShark: 2.75GB, Genozip: 2.59GB from original size 9.95GB in gz format) showed that methods such as GTShark, VCFShark, and GenoZip indeed achieved higher compression ratio for genotype compression (Results Section, Page 4 Line 110~114). Here we didn't include spVCF because we failed to compile the spVCF tool successfully in ubuntu:18.04 (the docker container). It should be noted that the compressed genotypes by GTShark, VCFShark, and Genozip cannot be randomly accessed without decompression, which will challenge the analysis speed, IO burden, and memory burden in large-scale genotype datasets.

As expected, GBC slightly sacrifices the compression ratio for high-performance accessibility. While a high compression ratio is important for data archive and transmission (as we pointed out in the Background Section Page 2 Line 42~44), the efficiency in accessing the compressed data is more critical for data analysis. GBC focus on the latter. Thus, to make it fair, we only compared tools that generated randomly accessible compressed genotypes.

**Q2:** In addition, the authors propose the compression of Genotypes rather than the whole VCF file. Note that while genotypes (and allele information) are the key information on VCF files, these files contained other information that should not be missed, such as quality metrics and annotations, among others. In order for the proposed method to see some real adoption, it is of utmost importance to include other components of the VCF file in the compressed file. Further, the proposed method should ideally be able to access these components efficiently (for example to help users efficiently filter the VCF files based on different quality metrics).

**Response:** We thank the reviewer for the constructive comments, which are related to Q6 by reviewer 1. In this new version, we have improved the structure of GTB to store other information (including all quality metrics of genotype) in VCF based on your great suggestions (http://pmglab.top/gbc/download/gbc-2.0.jar.zip). Please note that the core algorithm of GBC has been optimized for accessing compressed data (including genotypes and the quality metrics of genotypes). Meanwhile, GBC's access and file management performance is unaffected thanks to its bocking structure and optimization algorithms. Below are the Code Examples with `--addFields` (for input) and `--selectFields`(for output) options to contain other information.

| **GBC-2.0** | **http://pmglab.top/gbc/download/gbc-2.0.zip** |
| --- | --- |
| **Example** | **rare.disease.hg19.vcf.gz download from http://pmglab.top/gbc/download/example.zip** |
| # build GTB file, store INFO field and genotype metrics java -jar gbc-2.0.jar build ./example/rare.disease.hg19.vcf.gz \ --addFields GENOTYPE,INFO,META,GENOTYPE_METRIC --no-qc  # The following commands help the user to select which components in the VCF file to export and filter variants or genotypes by these fields: java -jar gbc-2.0.jar view ./example/rare.disease.hg19.gtb \ --selectFields GENOTYPE,GENOTYPE_METRIC \ --gty-gq 20 --gty-dp 4 --gty-ad 10 --gty-pl 20 --seq-qual 30 --seq-fs 60 --seq-mq 20 | |
| **Example** | **clinvar_20211225.vcf.gz download from**  **https://ftp.ncbi.nih.gov/pub/clinvar/vcf_GRCh37/archive_2.0/2022/clinvar_20211225.vcf.gz** |
| # build GTB file  java -jar gbc-2.0.jar build ./example/clinvar_20211225.vcf.gz \ --addFields INFO,META --no-qc  # extract data to the specified file  java -jar gbc-2.0.jar view ./example/clinvar_20211225.gtb ./example/clinvar_20211225.vcf | |
| **Example** | **rare.disease.hg19.vcf.gz download from http://pmglab.top/gbc/download/example.zip** |
| # build GTB file, store INFO field and genotype metrics java -jar gbc-2.0.jar build ./example/rare.disease.hg19.vcf.gz \ --addFields GENOTYPE,INFO,META,GENOTYPE_METRIC --no-qc  # The following commands help the user to select which components in the VCF file to export and filter variants or genotypes by these fields: java -jar gbc-2.0.jar view ./example/rare.disease.hg19.gtb \ --selectFields GENOTYPE,GENOTYPE_METRIC \ --gty-gq 20 --gty-dp 4 --gty-ad 10 --gty-pl 20 --seq-qual 30 --seq-fs 60 --seq-mq 20 | |
| **Example** | **clinvar_20211225.vcf.gz download from**  **https://ftp.ncbi.nih.gov/pub/clinvar/vcf_GRCh37/archive_2.0/2022/clinvar_20211225.vcf.gz** |
| # build GTB file  java -jar gbc-2.0.jar build ./example/clinvar_20211225.vcf.gz \ --addFields INFO,META --no-qc  # extract data to the specified file  java -jar gbc-2.0.jar view ./example/clinvar_20211225.gtb ./example/clinvar_20211225.vcf | |

minor comments:

**Q3:** Please do not use Chinese in the English version of the software website.

**Response:** We thank the reviewer for this comment. We have corrected this in the latest version of the software documentation (http://pmglab.top/gbc).

**Second round of review**

**Reviewer 2**

I appreciate the authors' effort to run the suggested methods for comparison. As expected (and as noted by the authors), these methods achieve better compression than the proposed method.

However, the authors discard further analysis of these method by stating that the compressed genotypes cannot be accessed without decompression. I understand that the genotypes in GBC, which are compressed by the z-standard (Fig. 1, e), would also need to be decoded prior to extracting the genotype, as the random access capabilities of GBC lay in the GTB file format structure. Further, the authors state in Fig. 2, e.: "This function of random access is only supported by BCFtools and GBC", but still compare in various panels of Fig. 2 against GTC, PBWT, BGT when assessing Random Access capabilities. Then, why not also compare against Genozip, GTShark and/or VCFShark?

Hence, the authors should include two of these methods in the complete assessment (Figure 2) to ensure a complete assessment of th proposed method. With out this comparison, one cannot fairly assess the state-of-the-art advancement of GBC. In any case, Fig. 2 has various panels where the newly analysed methods could be included even if not assessing their capabilities for random access.

Finally, I appreciate the authors' improvement of the the structure of GTB to stare other information from the VCF. I think it helps considerably with the potential adoption of the proposed method.

**Authors Response**

**Point-by-point responses to the reviewers’ comments:**

**Reviewer #2:**

I appreciate the authors' effort to run the suggested methods for comparison. As expected (and as noted by the authors), these methods achieve better compression than the proposed method.

However, the authors discard further analysis of these method by stating that the compressed genotypes cannot be accessed without decompression. I understand that the genotypes in GBC, which are compressed by the z-standard (Fig. 1, e), would also need to be decoded prior to extracting the genotype, as the random access capabilities of GBC lay in the GTB file format structure. Further, the authors state in Fig. 2, e.: "This function of random access is only supported by BCFtools and GBC", but still compare in various panels of Fig. 2 against GTC, PBWT, BGT when assessing Random Access capabilities. Then, why not also compare against Genozip, GTShark and/or VCFShark?

Hence, the authors should include two of these methods in the complete assessment (Figure 2) to ensure a complete assessment of th proposed method. With out this comparison, one cannot fairly assess the state-of-the-art advancement of GBC. In any case, Fig. 2 has various panels where the newly analysed methods could be included even if not assessing their capabilities for random access.

Finally, I appreciate the authors' improvement of the the structure of GTB to stare other information from the VCF. I think it helps considerably with the potential adoption of the proposed method.

**Response:** We thank the reviewer for these important comments. In this newly revised version, we compared the compression and data-access performance of Genozip and GTShark with GBC. Because VCFShark’s compression ratio, speed and decompression speed are very close to that of GTShark in genotypes, and it does not provide options for accessing compressed data, we skipped over VCFShark in the added performance evaluation.

While GTShark and Genozip have a slightly higher compression ratio than GBC for large-scale samples (say, GTShark 0.51GB, Genozip 0.48GB and GBC0.62GB for 469,835 subjects of 70,581 variants) (See more in Fig.2a, Supplementary material, Table S5 and Table S7), GBC outperforms GTShark and Genozip in terms of data-access speed by tens or even hundreds of times. The following are some representative comparison results about data-access speed. Fig2.e~Fig2.h and the Supplementary material (Table S5, S7, S9, and S10) show the detailed comparison results.

- **GTShark:** GTShark only supports extracting genotypes of a single subject from the compressed dataset. GTShark took 47,377.980 seconds to extract genotypes of a subject from a simulated genotype dataset of 5 million subjects and 100,000 variants, while GBC(v1.0) only needed 135.100 seconds.
- **Genozip:** Genozip can extract genotypes of multiple subjects or extract genotypes within a single genomic region from compressed datasets. In our tests, Genozip was 4576.7 times slower than GBC in accessing 9948 out of 99,448,478 variants in a dataset with 4,810 subjects (GBC’s 10.269s vs Genozip’s 46,998.596s). In a simulated genotype dataset with 500,000 subjects and 30,000 variants, Genozip was 845.89 times slower than GBC in accessing the genotypes of a subject(GBC’s 5.342s vs Genozip’s 4,518.747s). There were no comparsion results in the dataset with 5 million subjects because Genozip failed to compress the dataset with genotypes of 5 million subjects (throwing exception ERROR: genozip: Segconf didn't run because the first line is larger than 5000000).

Because Genozip and GTShark do not support file management functions (merging, splitting, and sorting by coordinates of variants), we did not compare these functions.

The file structure can explain why GBC has a much higher access speed than Genozip and GTShark. GBC’s file structure has a two-level index table for all genotype blocks and inside genotypes to allow quick searching. After locating the genotype blocks, only genotypes within the blocks are decompressed into the encoded genotypes (MBEG format). Moreover, only the needed part of the MBEG will be decoded into original genotypes. In contrast, Genozip and GTShark seemed to have no structure to facilitate access to compressed genotypes. However, we must state that the tools have different goals. Genozip and GTShark aim to achieve the best compression ratio for data archiving, while GBC is developed to primarily speed up the access of large-scale genotypes with a balance of the compression ratio.

Yes, it is right that the compressed genotypes cannot be directly accessed, and GBC also needs decompression before random access to genotypes. We also noted that the tools of GTC, PBWT and BGT had an option for accessing genotypes of variants in a given region, though not multiple ranges. The time of GTC, PBWT and BGT accessing multiple regions is equal to the summation of each region in the results section of the manuscript. Therefore, it is not accurate to state that "This function of random access is only supported by BCFtools and GBC". In the revised manuscript, we modified this sentence as "The option of accessing genotypes of variants in multiple regions at a time is only provided by BCFtools and GBC". And we also changed the "random access" to "genotype access" in the revised manuscript. We greatly appreciate your helpful comments on this issue.

**The following are details of the update in Figure 2.**

Fig.2a and Fig.2b: The comparison of compression ratio, compression speed, and decompression speed was performed uniformly for all tools using 469,835 UKBB subjects of a region on chromosome 4 (chr4: 51997648-190027301 of 70,581 variants), 2504 1000GP3 subjects (84,801,880 variants in the whole genome) and 4810 SG10K subjects (99,448,478 variants in the whole genome).

Fig.2c: We added compression and decompression speeds for Genozip and GTShark on simulated datasets to evaluate the performance of fast-access and data-archiving tools, respectively.

Fig.2d: No change.

Fig.2e, We added performance evaluation results of Genozip in accessing a set of random variants. However, Genozip failed to extract genotypes of 994,485 and 9,944,848 variants with the error message “ERROR: segmentation fault genocat -f -o -z 6 -@ 1 --regions-file”. GTShark has no option for accessing variants.

Fig.2f: We added the performance evaluation results of Genozip in accessing a set of consecutive variants. However, Genozip failed to complete this evaluation in the left panel because the process was aborted with datasets of more than 5 million subjects. Also, GTShark does not support this function.

Fig.2g, Fig.2h: We added performance evaluation results of Genozip and GTShark in accessing the genotypes of a set of subjects. Since GTShark can only extract one subjects at a time, we obtained the results of extracting multiple subjects by multiplying the time required to access a single subject.

Fig.2i: No change. Neither Genozip nor GTShark support filtering variants by allele count or allele frequency.

Fig.2j, Fig.2k, Fig.2l: No change.

Fig.2m, Fig.2n, Fig.2o, Fig.2p, Fig.2q: No change. Only GBC and BCFTools support direct file management on the compressed datasets (merging, splitting, and sorting by coordinates of variants).

**Third round of review**

**Reviewer 2**

While the authors have correctly address my concerns, I would suggest that they rephrase the wording to make it lass subjective. For example, please avoid the use of "The results show that there are no large differences" when the differences are almost 2x smaller, which in compression research is quite a lot (imagine reducing the burden of all genomic data by half....). In this regard, I would also recommend to change the titles of the section and remove the word "efficient".

**Authors Response**

**Point-by-point responses to the reviewers’ comments:**

Reviewer 2: While the authors have correctly address my concerns, I would suggest that they rephrase the wording to make it lass subjective. For example, please avoid the use of "The results show that there are no large differences" when the differences are almost 2x smaller, which in compression research is quite a lot (imagine reducing the burden of all genomic data by half....). In this regard, I would also recommend to change the titles of the section and remove the word "efficient".

Response: : We thank the reviewer for these important comments. We have altered the sentence “The results show that there are no large differences” to convey that ” The two methods’ compression ratios were around 1.7 to 1.27 times higher than that of GBC for unphased genotypes”.

Moreover, we have thoroughly reviewed and revised the manuscript to ensure a less subjective tone.

Additionally, we would like to emphasize that GBC is an efficient compression tool for large-scale genotypes as compared to its counterparts, such as GTC, PBWT, and BGT, which use block-based methods for fast accessing, characterised by the highest compression ratio and fastest compression speed (see Result section, Additional file 1: Table S1). Therefore, the term "efficient" is an apt description here.
